# Parameter uncertainty quantification using surrogate models applied to a spatial model of yeast mating polarization

**DOI:** 10.1371/journal.pcbi.1006181

**Published:** 2018-05-29

**Authors:** Marissa Renardy, Tau-Mu Yi, Dongbin Xiu, Ching-Shan Chou

**Affiliations:** 1 Department of Mathematics, Ohio State University, Columbus, Ohio, United States of America; 2 Department of Molecular, Cellular, and Developmental Biology, University of California, Santa Barbara, California, United States of America; Johns Hopkins University, UNITED STATES

## Abstract

A common challenge in systems biology is quantifying the effects of unknown parameters and estimating parameter values from data. For many systems, this task is computationally intractable due to expensive model evaluations and large numbers of parameters. In this work, we investigate a new method for performing sensitivity analysis and parameter estimation of complex biological models using techniques from uncertainty quantification. The primary advance is a significant improvement in computational efficiency from the replacement of model simulation by evaluation of a polynomial surrogate model. We demonstrate the method on two models of mating in budding yeast: a smaller ODE model of the heterotrimeric G-protein cycle, and a larger spatial model of pheromone-induced cell polarization. A small number of model simulations are used to fit the polynomial surrogates, which are then used to calculate global parameter sensitivities. The surrogate models also allow rapid Bayesian inference of the parameters via Markov chain Monte Carlo (MCMC) by eliminating model simulations at each step. Application to the ODE model shows results consistent with published single-point estimates for the model and data, with the added benefit of calculating the correlations between pairs of parameters. On the larger PDE model, the surrogate models allowed convergence for the distribution of 15 parameters, which otherwise would have been computationally prohibitive using simulations at each MCMC step. We inferred parameter distributions that in certain cases peaked at values different from published values, and showed that a wide range of parameters would permit polarization in the model. Strikingly our results suggested different diffusion constants for active versus inactive Cdc42 to achieve good polarization, which is consistent with experimental observations in another yeast species *S. pombe*.

## Author summary

Mathematical models in systems biology often have many parameters, such as biochemical reaction rates, whose true values are unknown. When the number of parameters is large, it becomes computationally difficult to analyze their effects and to estimate parameter values from experimental data. This is especially challenging when the model is expensive to evaluate, which is the case for large spatial models. In this paper, we introduce a methodology for using surrogate models to drastically reduce the cost of parameter analysis in such models. By using a polynomial approximation to the full mathematical model, parameter sensitivity analysis and parameter estimation can be performed without the need for a large number of model evaluations. We explore the application of this methodology to two models for yeast mating polarization. A simpler non-spatial model is used to demonstrate the techniques and compare with published results, and a larger spatial model is used to demonstrate the computational savings offered by this method.

## Introduction

Mathematical models provide a more quantitative description of biological systems compared to qualitative arrow diagrams. A major tool of mathematical modeling is differential equations representing the dynamics of various components of the system which may be a cell, organism, or ecosystem [1–3]. In models describing cellular dynamics, the components are typically different protein species, and their changing levels depend on the biochemical reactions between the species [4, 5]. If spatial dynamics, such as diffusion and advection, are present and considered, partial differential equation (PDE) models must be used; otherwise, the system can be modeled by ordinary differential equations (ODE).

One of the challenges in modeling is identifying the parameters from data [6, 7]. For cellular models these parameters include the kinetic rate constants in the various reaction terms, as well as initial conditions of the modeled species. Two important tools of parameter analysis are sensitivity analysis and parameter estimation. Parameter sensitivity analysis is used to quantify the degree to which each parameter affects an output of interest. Two general types of sensitivity analysis, local and global sensitivity analysis, have been widely used. In local sensitivity analysis, sensitivities are evaluated at a single parameter set, whereas in global sensitivity analysis, sensitivities are evaluated across the entire parameter space. These analyses have been widely applied, such as to models in epidemiology [8–12], signalling pathways [13–15], physiology [16], and wound healing [17].

For parameter estimation, two major approaches are Bayesian and maximum likelihood [7, 18]. The primary difference between these two is that Bayesian methods infer a probability distribution for the parameters based on the available data, whereas maximum likelihood methods will provide a single-point estimate. In systems biology, parameter estimation has been widely applied, via both Bayesian inference [11, 19–21] and optimization methods [22–25]. An important advantage of the Bayesian approach is a more explicit representation of the uncertainty in the parameter estimates; however, that usually comes at a price of high computational cost for estimating the distribution by extensive sampling.

In general, global sensitivity analysis and parameter estimation both require sampling of the parameter space. For systems with large parameter counts, this can become very challenging due to the curse of dimensionality. Too many parameters can make sampling of the parameter space computationally intractable, especially for partial differential equation models that are expensive to solve. Many advances have been made in reducing computational cost in the field of uncertainty quantification (UQ), which is concerned with the characterization and reduction of uncertainty in mathematical models [26–28]. Polynomial approximation has proven to be a key tool in uncertainty quantification. System outputs can be approximated by an orthogonal polynomial expansion (polynomial chaos), allowing for straight-forward computation of statistical quantities [29].

In this paper, we apply a method for parameter sensitivity analysis and parameter estimation that uses polynomial approximation to significantly reduce the computational cost for large problems. A key step in the proposed method is the construction of a polynomial surrogate model. This surrogate model allows for sampling methods to be applied without the need to solve the full system for each sample. The use of surrogate models (e.g. support vector machines) for biological systems has been explored previously in [16], and the use of polynomial approximations for uncertainty analysis has been investigated in [30, 31].

To demonstrate the capability of the proposed method, we apply it to models of yeast cell polarization. Cell polarization is the process by which intracellular species (e.g. proteins) become asymmetrically localized, which is fundamental to cellular processes such as cell division, differentiation, and movement [32, 33]. Failure in polarization can lead to cell death or dysfunction, and abnormal cell polarity is characteristic of cancer and may contribute to tumor initiation [34]. Cell polarization has been extensively studied in the budding yeast *S. cerevisiae* due to its pronounced polarity and genetic tractability [35, 36]. The models analyzed in this paper describe polarization in response to pheromone during mating in budding yeast.

We consider two models: an ODE model for only one module of the system (the heterotrimeric G-protein cycle), and a spatial model that incorporates a larger signaling pathway as well as membrane diffusion of the proteins. We will refer to these models as Model 1 and Model 2, respectively. Model 1 was proposed in [37] and has eight kinetic rate parameters, six of which have been experimentally measured or approximated from the literature. The remaining two parameters were estimated in [37] via an optimization method. This model is used to demonstrate the method and for comparison with the previous results. Model 2 is a mechanistic reaction-diffusion model, which is an extension of the model considered in [38]. This model has 35 unknown parameters. Parameter sensitivity analysis and parameter estimation have not previously been performed for this model, in part due to the large number of parameters. We seek to utilize polynomial surrogate models to quantify the effects of the parameters on polarization and to infer the biologically reasonable parameter values.

It should be noted that the results of parameter sensitivity and parameter estimation are dependent on the assumed model structure. In systems biology there is often significant uncertainty in the model structure itself. Some work has been done on quantifying the structural uncertainty in models of biological networks and reconstructing networks from data [39–41]. However, this is beyond the scope of the present work and this source of uncertainty is not addressed in this paper.

The structure of this paper is as follows. We first present the mathematical methods for surrogate model construction and how to perform parameter sensitivity analysis and parameter estimation using a polynomial surrogate. We then demonstrate the methods on Model 1, performing sensitivity analysis and estimation in two cases: first, varying only the two free parameters, and second, varying all eight parameters. We then present Model 2 and use sensitivity analysis to significantly reduce the parameter count. Bayesian parameter estimation is then performed in the reduced parameter space. We discuss the computational savings afforded by the use of a polynomial surrogate for parameter estimation in Model 2. Finally, we discuss biological implications of the results and future applications of the polynomial surrogates in Bayesian model analysis.

## Methods

### Surrogate model construction

Biological systems often possess many parameters whose true values are unknown. In order to gain an understanding of the effects of each parameter, we need to sample the parameter space. However, sampling a high-dimensional space is a difficult task. For example, in the next section we consider a large PDE model with 35 parameters. In this case, even with only two sample points in each dimension we would need 2^35^ ∼ *O*(10^10^) samples, and each sample requires solving a PDE system. This makes direct sampling of the PDE impractical. Instead, we may choose a scalar response function that quantifies an output of interest and by assuming that this response function depends smoothly on the parameters, a polynomial can be fit using far fewer sample points. Since we are performing parameter estimation, the response function depends not only on what quantity is of interest but also on what experimental data are available.

If multiple response functions are of interest (for example, different time points or different values of some input), there are two options—one can either increase the number of variables in the polynomial or use multiple polynomials. For example, if measurements are taken at several time points *t*_1_,…,*t*_*k*_, then either *t* may be introduced as a variable of the polynomial or a polynomial *P*_*i*_ can be fit for each time point (*i* = 1,…, *k*). The choice can be made based on computational cost. If data are sparse, it is usually best to fit multiple polynomials, which is the approach taken in this work. Once the polynomial is established, we can use it as a surrogate for the full model so that sampling of the parameter space is far less expensive.

To perform the polynomial fitting, we use an orthogonal polynomial basis from the generalized polynomial chaos (gPC) approach [26, 29]. Thus the choice of basis for the polynomial space depends on the assumed probability distribution of the parameters. For the examples considered in this work, we assume that the parameters are independent and identically distributed, and uniformly distributed in a fixed range. This leads to the use of a Legendre polynomial basis. All parameters are mapped to a standard reference interval of [−1, 1]. We do not consider any other distributions, but the same principles can be applied if the parameters have a Gaussian distribution (Hermite polynomials), Gamma distribution (Laguerre polynomials), or Beta distribution (Jacobi polynomials).

Recall that the number of basis functions for the set of polynomials of degree up to *d* in *n* variables is $\left(\begin{array}{c} n+d\\ n \end{array}\right)$. The polynomial coefficients can be solved for in a number of ways, depending on the number of samples available. If the number of samples is exactly $\left(\begin{array}{c} n+d\\ n \end{array}\right)$, then the coefficients can be solved for by direct interpolation. This case should generally be avoided as interpolation is notoriously prone to instability. If the number of samples is greater than $\left(\begin{array}{c} n+d\\ n \end{array}\right)$, least squares approximation can be used. If the number of samples is less than $\left(\begin{array}{c} n+d\\ n \end{array}\right)$, which is the case of interest for large problems, one may use compressed sensing methods to solve for the coefficients [42]. This approach has been well established for UQ problems [43, 44].

The samples can be chosen in a variety of ways (e.g. uniform random sampling, sparse grids, Latin hypercube sampling, etc.). A quasi-optimal sampling scheme for least squares polynomial fitting has been explored in [45]. In the applications presented here, we use uniform random sampling. Details of the polynomial fitting are presented in Algorithm 1.

**Algorithm 1** Polynomial fitting algorithm.

1. Determine the desired polynomial degree and how many samples can reasonably be obtained.

2. Sample the parameter space using the sampling method of your choice. The sampling method may depend on whether you are undersampling or oversampling (e.g. for oversampling, you may want to use quasi-optimal points for least squares [45]).

3. Using the samples from step 2, set up a linear system *Ax* = *b* where *x* is the vector of polynomial coefficients, *A* is a matrix whose entries are the basis polynomials evaluated at the sample points (each row corresponds to one sample, each column corresponds to one basis polynomial), and *b* is a column vector of the model output at the sample points.

4. Solve for the coefficients. If undersampling, perform compressed sensing with *ℓ*_1_-minimization. If oversampling, perform least-squares fitting.

The accuracy of the polynomial can be estimated by cross-validation. In cross-validation, the model is evaluated at additional sample points that were not used in the polynomial fitting. The model output can then be compared with the polynomial value at those points to determine the error. One may also perform *k*-fold cross validation in which the total set of sample points is partitioned into *k* equally sized subsets; call them Ω_*i*_, *i* = 1,…, *k*. Cross-validation is then performed *k* times. For each *i*, the samples in Ω_*i*_ are used to evaluate the error and the remaining samples are used to fit the polynomial. The acceptable level of error will depend on the particular application.

### Parameter sensitivity analysis and parameter estimation

**Fig 1:**
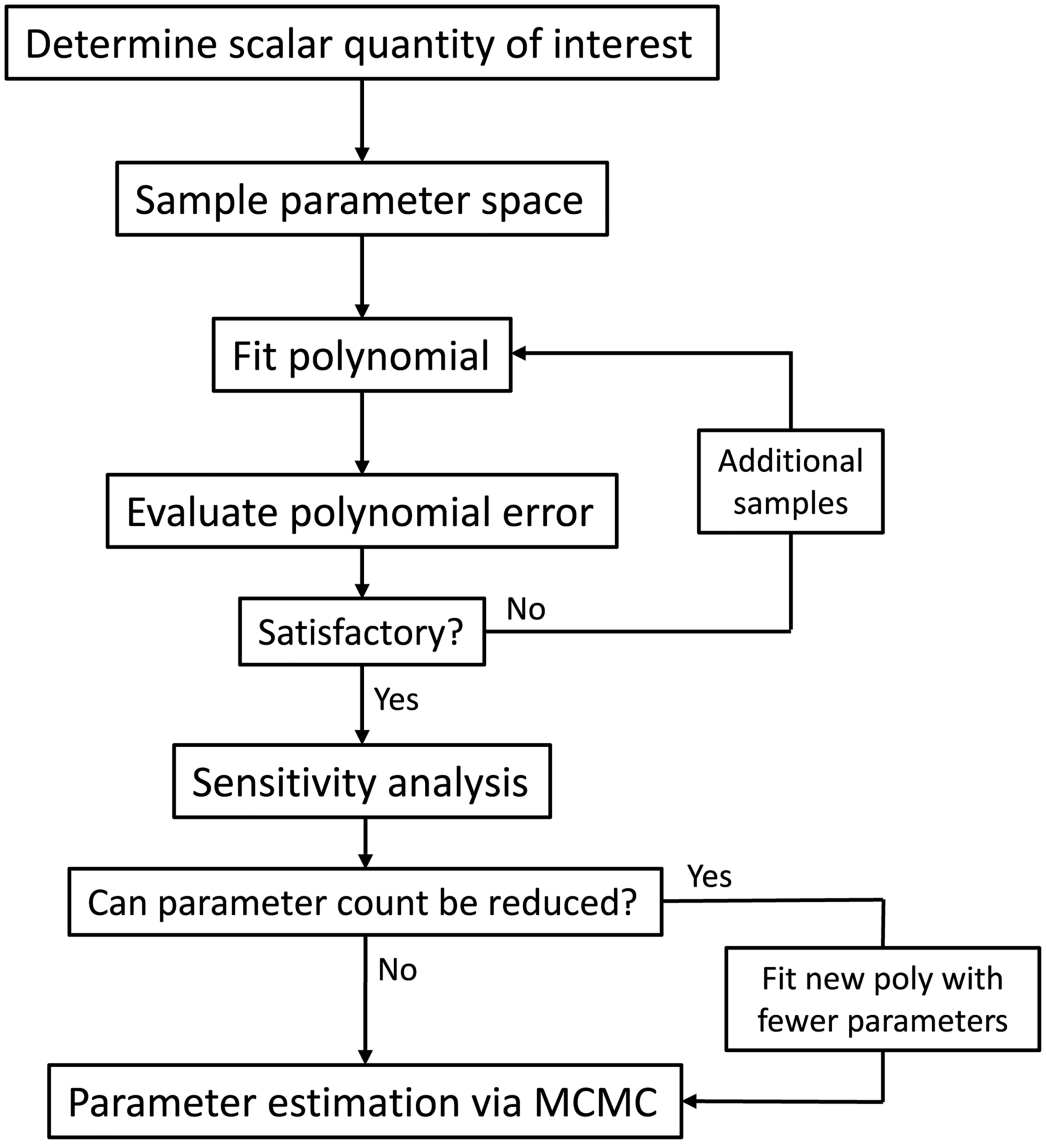
Flowchart for parameter analysis described in methods. The polynomial fitting procedure is described in Algorithm 1. The parameter space samples are generated by model simulation. The sensitivity analysis and parameter estimation use the fitted surrogate polyomial.

Once the polynomial surrogate model is constructed, it can be used to perform parameter sensitivity analysis and parameter estimation (Fig 1). Any sensitivity or estimation method can be applied using the polynomial surrogate model to decrease computational cost. In the work presented here, the methods are as follows.

We define the sensitivity of a response function *z*(*p*_1_,…, *p*_*n*_) to a parameter *p*_*j*_ as $S_{j}=E(\frac{\partial z}{\partial p_{j}})$. We refer to *S*_*j*_ as the sensitivity coefficient for *p*_*j*_. Note that, while the partial derivative is typically used for local sensitivity analysis, the expectation makes this a global measure of sensitivity since $\frac{\partial z}{\partial p_{j}}$ is integrated over the entire parameter space. Using the surrogate model, the parameter sensitivities can be analytically computed by taking partial derivates and evaluating $S_{j}=\int \frac{\partial z}{\partial p_{j}}d\rho$, where *ρ* is the probability measure associated with the *n*-dimensional parameter space.

We can then assess the importance of each parameter based on its sensitivity. If the response is not sensitive to a parameter *p*_*j*_, then the dynamics of the model will likely remain unchanged if *p*_*j*_ is fixed. Further, *p*_*j*_ may be non-identifiable so that multiple values can produce an equally good fit to data. Thus, we may use the sensitivity analysis to decrease the parameter count by fixing those parameters that have small sensitivity coefficients.

For parameter estimation, we use Markov chain Monte Carlo (MCMC) method with Metropolis-Hastings algorithm [46]. MCMC is a method for sampling the posterior distribution of the parameters—that is, the parameter distribution that corresponds to the distribution of the provided data, given an assumed prior distribution. For the prior distribution, we use the parameter distribution that was assumed in the construction of the surrogate polynomial (in this case, uniformly distributed within a range). This Bayesian approach to parameter estimation provides both the most probable parameter set (or sets) as well as a characterization of the parameter uncertainty.

MCMC methods have become a popular choice for parameter estimation in biological systems [21, 47, 48]. However, these methods are often prohibitively expensive for computationally intensive models, since each sample in the Markov chain requires a model evaluation. By using the polynomial surrogate, the cost is greatly reduced. Further, it has been shown that in the generalized polynomial chaos framework, the polynomial fit and the resulting posterior distribution have similar convergence properties [49]. Thus, if the error in the polynomial fit is small, we expect the error in the posterior distribution to also be small.

A key question is knowing when the MCMC has converged, meaning that the distribution of the Markov chain samples has converged to the posterior distribution. Several convergence diagnostics for MCMC have been proposed [50, 51]. We employ a simple test which is to run multiple Markov chains from different initial parameter sets and compare the resulting distributions. Roughly speaking, if the independent chains stabilize at the same distribution, then the MCMC has converged. Since the chains are independent, they can be run in parallel to save computing time. We choose MCMC over alternative sampling methods [20, 52, 53] because of its efficiency. Since MCMC is based on a Markov chain, the samples tend toward higher probability areas of the parameter space in contrast to schemes that may sample the entire space.

All codes have been made publicly available on GitHub in the repository https://github.com/chingshanchou/UQ-Yeast-Mating-Model.

### Experimental techniques

The yeast strain CGY-021 is a derivative of W303-1A and contains the *bar1*Δ mutation that prevents *α*-factor degradation by deletion of the Bar1 protease. GFP has been integrated genomically at the C-terminus of Ste20 to create a Ste20-GFP fusion protein that is a fluorescent reporter for active Cdc42 [54]. The genotype of the strain CGY-021 is *MAT**a***, *can1-100*, *ade2-1*, *leu2-3*,-*112*, *his3-11*,-*15*, *trp1-1*, *ura3-1*, *bar1::hisG*, *ste20*Δ*::STE20-GFP-HIS5*.

Cells were cultured in YPD (yeast extract-peptone-dextrose) media supplemented with adenine. Cells were treated for 60 minutes with 10 nM *α*-factor and then fixed with formaldehyde. Visualization was performed using a 60x objective (NA = 1.4) on an Olympus Fluoview 1000 Spectral confocal microscope. The resulting images were analyzed in Matlab and the membrane fluorescent intensity was quantified over the periphery of the cell to generate the polarization profile that was averaged over 20 cells and converted into a polarization factor value.

## Results

We apply the proposed method to two models of the yeast mating response. Haploid budding yeast cells assume two mating types, **a** or *α*, and each secretes a pheromone (**a**-factor and *α*-factor, respectively) to attract the opposite type. We consider the response of an **a**-cell to *α*-factor, although the two cases are nearly identical [55]. During the mating process, *α*-factor binds and activates the *α*-factor receptors on the **a**-cell surface, which leads to a sequence of reactions that results in the recruitment of polarization proteins to the cell membrane, causing the formation of the mating projection. These reactions occur in two G-protein cycles (unidirectional cycles formed by the activation and deactivation of G-proteins) which are the heterotrimeric and Cdc42 G-protein cycles [36, 56, 57]. A description of these cycles can be found in [38], and are summarized below.

First, the pheromone *α*-factor (L) binds the *α*-factor receptor (R) to form the receptor-ligand complex (RL). This activates the heterotrimeric G-protein (G) to form active *α*-subunit (Ga) and free G*βγ* (Gbg). G*α* can be deactivated to form inactive *α*-subunit (Gd), which binds G*βγ* to reform G. The free G*βγ* then recruits Cdc24 (C24) to the cell membrane (C24m), which activates Cdc42 (C42) to its active form (C42a). Active Cdc42 then binds the scaffold protein Bem1 (B1) and recruits it to the membrane (B1m). Membrane-bound Bem1 is then able to recruit more Cdc24 to the membrane, creating a positive feedback loop. In addition, active Cdc42 turns on the kinase Cla4 (Cla4a), which inhibits Cdc42 activation by negatively regulating Cdc24. The pathway is summarized in the schematic diagram in S1 Fig.

Two key features of this process are the positive and negative feedback loops. In the positive feedback loop, membrane-bound Bem1 binds and activates Cdc24 which catalyzes the formation of active Cdc42 which binds more Bem1. In the negative feedback loop, active Cdc42 activates Cla4 which inhibits the membrane-bound Cdc24, leading to a lower activation rate of Cdc42. Cdc42 is of particular interest since it plays a key role in establishing polarity and is highly conserved from yeasts to humans [58].

### Model 1: The heterotrimeric G-protein cycle

To demonstrate our methods, we first consider a simple model: an ODE model of the heterotrimeric G-protein cycle taken from [37]. These equations represent the first stage of the system that senses the input ligand (L) *α*-factor:

$$\frac{d[R]}{dt}=-k_{RL}\left[L\right]\left[R\right]+k_{RLm}\left[RL\right]-k_{Rd0}\left[R\right]+k_{Rs},$$ (1)

$$\frac{d[RL]}{dt}=k_{RL}\left[L\right]\left[R\right]-k_{RLm}\left[RL\right]-k_{Rd1}\left[RL\right],$$ (2)

$$\frac{d[G]}{dt}=-k_{Ga}\left[RL\right]\left[G\right]+k_{G1}\left[Gd\right]\left[Gbg\right],$$ (3)

$$\frac{d[Ga]}{dt}=k_{Ga}\left[RL\right]\left[G\right]-k_{Gd}\left[Ga\right],$$ (4)

where the *k*’s are reaction rates. Here, [*Gd*] = *Gt* − [*G*] − [*Ga*] and [*Gbg*] = *Gt* − [*G*], with *Gt* being the total number of G-protein molecules per cell. The model output is the fraction of free G*βγ* (*Gbg*/*Gt*), and the time unit is seconds. The model contains 9 parameters (8 rate constants and *Gt*), 7 of which were determined in [37] from experimental measurements and information from the literature. The remaining two parameters (*k*_*Ga*_ and *k*_*Gd*_) were fit to data in [37] via least squares minimization. These parameter values are given in S1 Table. We focus first on this two-parameter problem, and use the proposed methods to corroborate the published parameter estimates. Later, we will allow all eight kinetic parameters to vary to determine if the same parameter estimates are obtained in the larger parameter space. In the 2-dimensional sensitivity analysis and parameter estimation, we will assume that the parameters *k*_*Ga*_ and *k*_*Gd*_ are log-uniformly distributed in the intervals [10^−7^, 10^−3^] and [10^−3^, 10], respectively, which span the relevant ranges for the parameters.

#### Construction of the polynomial surrogate model

Since the ultimate goal is parameter estimation, the response functions of interest are those outputs for which we have experimental data. Using the data from [37], we have measurements of the free G*βγ* fraction (*Gbg*/*Gt*) at the time points and *α*-factor levels specified in S2 Table.

We first construct a polynomial surrogate model that approximates the ODE model which allows us to sample the parameter space at a much lower computational cost. In this example, we construct a set of polynomials in two variables (*k*_*Ga*_ and *k*_*Gd*_), whose output represents *Gbg*/*Gt* for the time-course and dose-response data. Since the dimension of the polynomial space is relatively low, we can perform polynomial fitting by least squares approximation. To fit the polynomial, we first map the parameter ranges to [−1, 1], and then fit the polynomial using a Legendre basis (see Methods).

The degree of the polynomial as well as the number of points used for least squares fitting can be adjusted depending on the error of the resulting polynomial. The error can be determined by calculating the difference between the polynomial and the simulated full model at randomly sampled points using cross-validation. Since the number of samples may need to be adjusted, it is best to use a sampling technique that allows for the sequential addition of points, such as simple random sampling or Sobol sampling.

**Fig 2:**
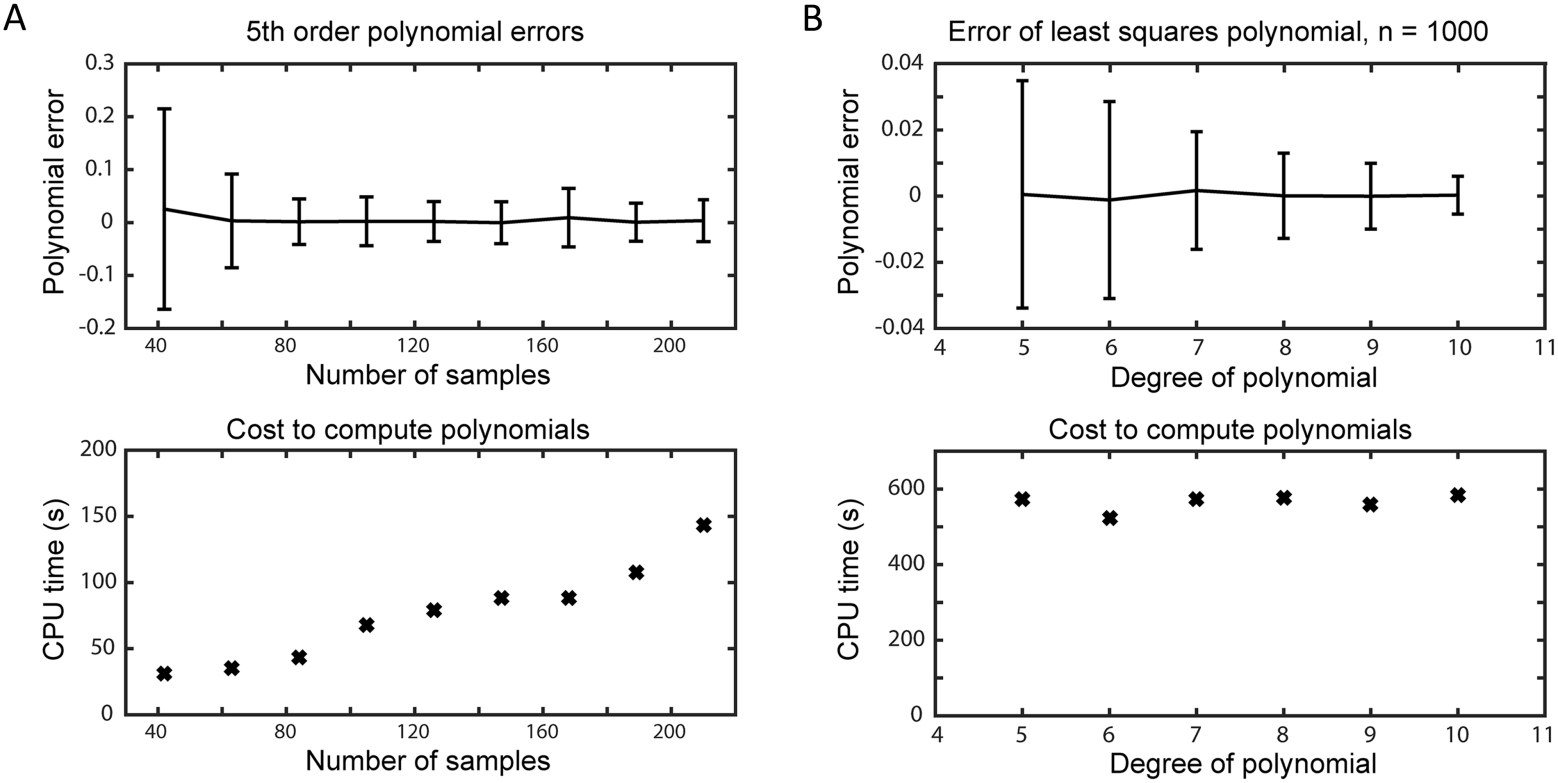
Polynomial errors. Error mean and standard deviation (measured using 100 random samples by cross-validation) for different polynomial fits (top), and the cost to compute the polynomials (bottom). (A) 5th order polynomials fit using different numbers of sample points. (B) Polynomials of varying degree using least squares fitting with 1000 points. Polynomial error is the average difference between the polynomial and the model output, and the error bars indicate the standard deviation of the error over the 100 sample points.

In Fig 2A, we use uniform random sampling to fit a 5th order polynomial. The error of the polynomial stabilizes quite rapidly with the addition of more sample points, and settles to a roughly constant standard deviation (with a mean near 0) when the number of sample points is 4 times the number of basis polynomials. In Fig 2B, we fit polynomials of varying degree (up to degree 10) using 1000 sample points. With this relatively large sample size, the standard deviation of the polynomial error decreases as the polynomial degree grows. Thus, the polynomial can be made more accurate by increasing the number of samples and the polynomial degree. It should be noted that for a small sample size, increasing the degree does not necessarily improve the polynomial error due to extrapolation error and Runge’s phenomenon [59].

We plot the computational cost of the polynomial fitting as a function of number of samples or polynomial degree at the bottom of Fig 2. We find that the cost is primarily determined by sample size.

#### Sensitivity analysis reveals importance of *k*_*Ga*_ and *k*_*Gd*_

For the sensitivity analysis and parameter estimation, we use the 10th degree polynomial fit from 1000 sample points. Since each data point in S2 Table acts as its own response function, we obtain 15 sensitivity coefficients for each parameter (one per response function). These coefficients are given in Table 1 along with their means which give an idea of the overall sensitivity of the system to the parameters *k*_*Ga*_ and *k*_*Gd*_. We observe from the sensitivity coefficients that a larger value of *k*_*Ga*_ is associated with a larger value of *Gbg*/*Gt* for all of the data points, and a larger value of *k*_*Gd*_ is associated with a smaller value of *Gbg*/*Gt*. Interestingly *k*_*Ga*_ has a larger effect on the output than *k*_*Gd*_, but both effects are substantial (magnitude between 0.1 and 1), and the sensitivities of the data points are close to their mean.

**Table 1:** Sensitivities of output (*Gbg*/*Gt*) to *k*_*Ga*_ and *k*_*Gd*_ based on a 10th degree polynomial fit. Sensitivity coefficients are given for different time points (*T*, secs) and *α*-factor concentrations (*L*, nM), as well as the overall mean.

Data points		Sensitivity to *k*_*Ga*_	Sensitivity to *k*_*Gd*_
*L* = 1000	*T* = 10	4.6 × 10^−1^	−2.4 × 10^−1^
*L* = 1000	*T* = 30	4.5 × 10^−1^	−3.0 × 10^−1^
*L* = 1000	*T* = 60	4.5 × 10^−1^	−3.4 × 10^−1^
*L* = 1000	*T* = 120	4.3 × 10^−1^	−3.7 × 10^−1^
*L* = 1000	*T* = 210	4.3 × 10^−1^	−4.0 × 10^−1^
*L* = 1000	*T* = 300	4.2 × 10^−1^	−4.0 × 10^−1^
*L* = 1000	*T* = 450	4.0 × 10^−1^	−4.0 × 10^−1^
*L* = 1000	*T* = 600	3.9 × 10^−1^	−4.0 × 10^−1^
*L* = 1	*T* = 60	3.5 × 10^−1^	−2.0 × 10^−1^
*L* = 2	*T* = 60	3.8 × 10^−1^	−2.3 × 10^−1^
*L* = 5	*T* = 60	4.2 × 10^−1^	−2.7 × 10^−1^
*L* = 10	*T* = 60	4.4 × 10^−1^	−2.9 × 10^−1^
*L* = 20	*T* = 60	4.4 × 10^−1^	−3.1 × 10^−1^
*L* = 50	*T* = 60	4.4 × 10^−1^	−3.2 × 10^−1^
*L* = 100	*T* = 60	4.5 × 10^−1^	−3.2 × 10^−1^
Mean sensitivity		4.2 × 10^−1^	−3.2 × 10^−1^

#### Parameter estimation of *k*_*Ga*_ and *k*_*Gd*_ is in agreement with previous estimates

**Fig 3:**
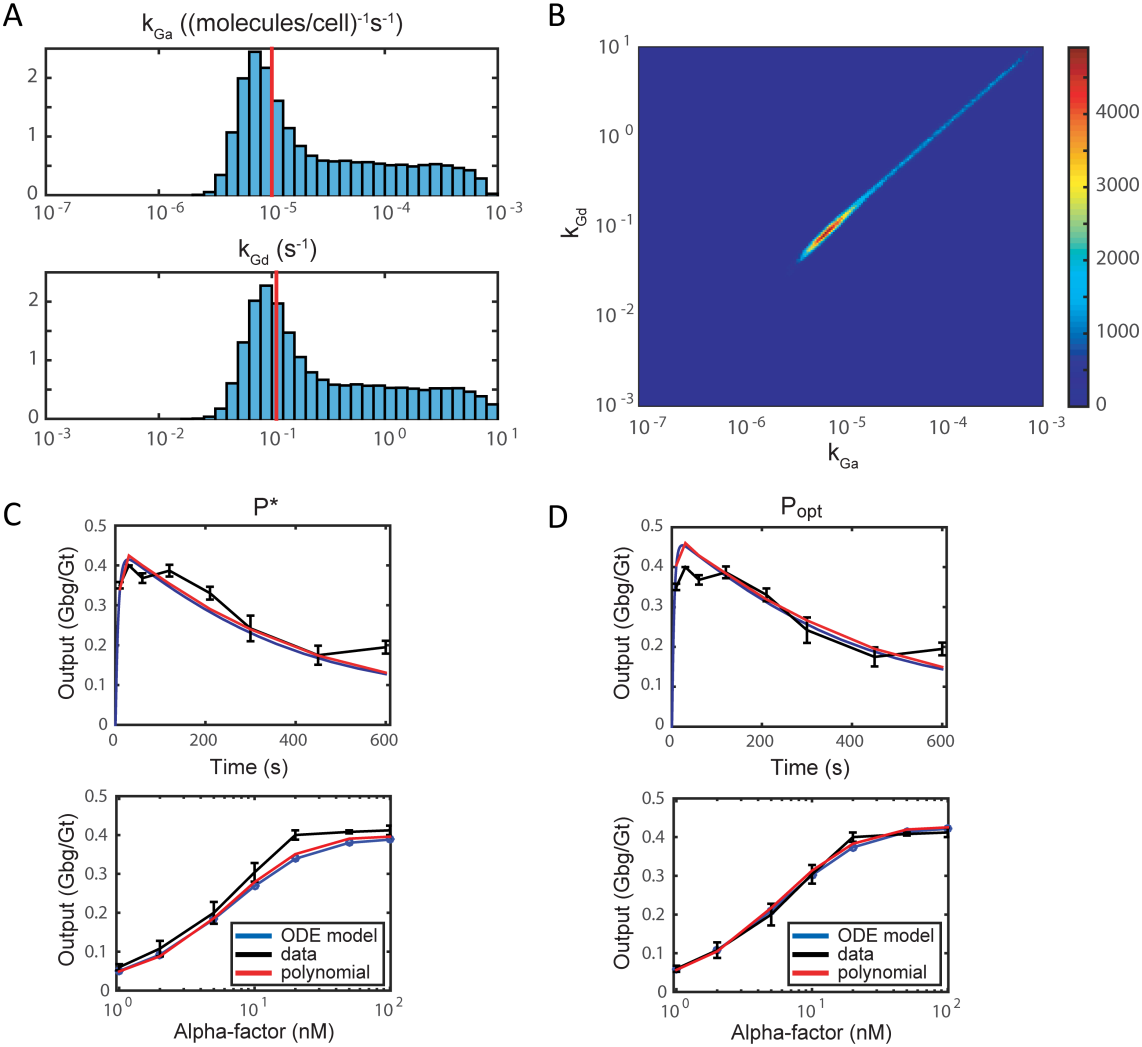
Parameter estimation of *k*_*Ga*_ and *k*_*Gd*_ in ODE model (1)–(4). Probability distributions are obtained via Markov chain Monte Carlo and a 10th degree polynomial. (A) Distributions for individual parameters, normalized so that the total area is equal to 1. Red lines indicate the optimal (maximum likelihood) parameter values *P*_*opt*_. (B) Colormap of the two-dimensional joint probability distribution of *k*_*Ga*_ and *k*_*Gd*_ from the MCMC chain. Red indicates high probability along the diagonal; blue indicates low prsobability. (C, D) Model (blue) and polynomial (red) outputs corresponding to parameter sets P* and *P*_*opt*_, respectively, compared with the data (black) from [37] for the time-course (top) and dose-response (bottom) experiments.

We perform parameter estimation using the data from [37] and described in S2 Table. Markov chain Monte Carlo with a 10th degree polynomial surrogate generates the parameter distributions shown in Fig 3A and 3B over a range of four orders of magnitude. The length of the Markov chain is 10^6^ steps with a burn-in period of 10^5^ steps. The distribution has a single mode at $P^{*}=\left(k_{Ga}^{*},k_{Gd}^{*}\right)=\left(7.1\times 10^{-6},9.0\times 10^{-2}\right)$, which is close to the optimal (maximum likelihood) estimate *P*_*opt*_ = (1 × 10^−5^, 1.1 × 10^−1^) from [37]. The units for *k*_*Ga*_ and *k*_*Gd*_ are (molecules per cell)^−1^s^−1^ and s^−1^, respectively. Simulating the model with P* produces a good fit to the time-course and dose-response data shown in Fig 3C and 3D. The mean squared errors for P* is 4.1 × 10^−4^, while the mean squared error for *P*_*opt*_ is 1.3 × 10^−4^. Finally, plotting the two-dimensional distributions of *k*_*Ga*_ and *k*_*Gd*_ show that they are highly correlated with the highest probability region lying along the diagonal (Fig 3B).

#### Allowing all 8 parameters to vary produces parameter estimates consistent with published values

We now apply the same parameter estimation procedure to the G-protein model allowing all 8 of the kinetic parameters to vary. In other words, we assume that all the parameters are unknown and would like to use our model to estimate these parameters. The parameters are assumed to be log-uniformly distributed in the ranges in S3 Table. All parameter ranges span two orders of magnitude (the ranges for *k*_*Ga*_ and *k*_*Gd*_ are shrunk from the previous section but still contain P* and *P*_*opt*_).

For this problem we choose a 5th degree polynomial surrogate that allows oversampling; the 5th degree polynomial space in 8 parameters has 1287 basis polynomials. We perform uniform random sampling on 1500 points generated by model simulation to construct the polynomial by least squares fitting. The resulting polynomial has mean absolute error 2.5 × 10^−2^.

Using the polynomial as a surrogate for the full model, we compute parameter sensitivities for the 8 parameters, and the mean sensitivities over the dataset are given in Table 2. As expected, we observe that the output (*Gbg*/*Gt*) is most sensitive to *k*_*Ga*_ and *k*_*Gd*_ whose sensitivities decreased slightly compared to when the other 6 parameters are fixed.

**Table 2:** Sensitivities of the ODE model output (*Gbg*/*Gt*) to all 8 kinetic parameters using a 5th degree surrogate polynomial. Both mean sensitivities and the mean of the absolute value of the sensitivities are shown.

Parameter	Mean sensitivity	Mean abs. value of sensitivity
*k*_*RL*_	8.2 × 10^−2^	8.2 × 10^−2^
*k*_*RLm*_	−3.2 × 10^−2^	3.2 × 10^−2^
*k*_*Rs*_	9.2 × 10^−3^	1.2 × 10^−2^
*k*_*Rd*0_	1.1 × 10^−3^	6.3 × 10^−3^
*k*_*Rd*1_	−6.2 × 10^−2^	6.2 × 10^−2^
*k*_*G*1_	5.6 × 10^−4^	7.4 × 10^−3^
*k*_*Ga*_	3.1 × 10^−1^	3.1 × 10^−1^
*k*_*Gd*_	−2.6 × 10^−1^	2.6 × 10^−1^

**Fig 4:**
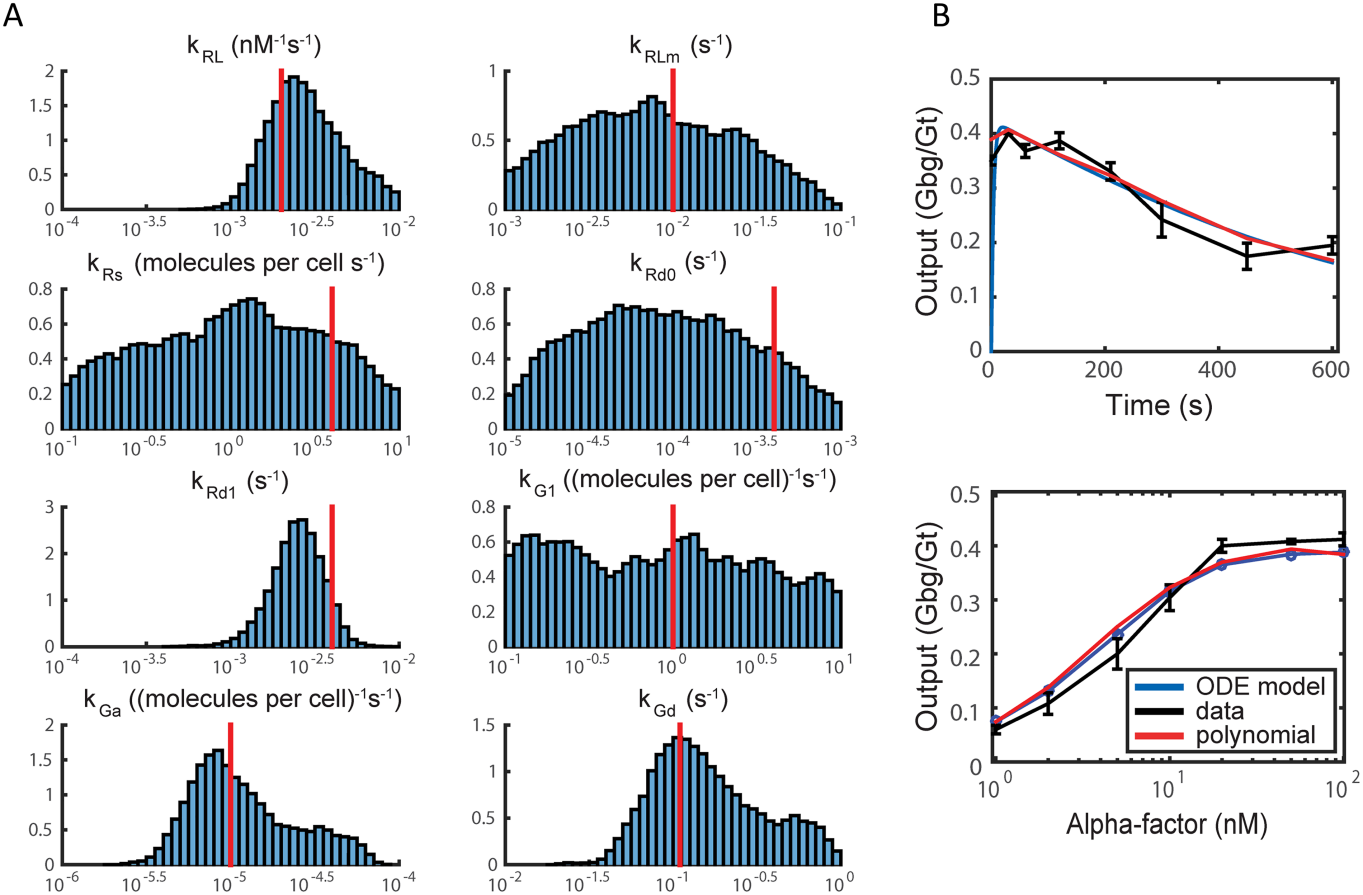
MCMC results for ODE model (1)–(4). (A) Parameter distributions from ODE model (1)–(4) for all 8 kinetic parameters obtained via MCMC. Red vertical lines indicate the parameter values from experiments or maximum likelihood estimates [37]. Markov chain length was 10^6^ steps. (B) Model simulation and polynomial outputs using the mean parameter set from the 8-parameter MCMC compared with the time-course (top) and dose-response (bottom) data.

Next, we perform parameter estimation on all 8 parameters and obtain the distributions in Fig 4A. The distributions have a single relatively sharp peak for 4 parameters (*k*_*RL*_, *k*_*Rd*1_, *k*_*Ga*_, and *k*_*Gd*_), which correspond to the parameters with the highest sensitivities. The peak values in these cases are close to the measured or estimated parameter values from [37]. The other 4 parameters possess broader distributions with the distribution for heterotrimeric G-protein reassociation (*k*_*G*1_) being nearly flat consistent with its low sensitivity. The values of the rate constants for receptor synthesis (*k*_*Rs*_) and degradation (*k*_*Rd*0_, *k*_*Rd*1_) are somewhat lower than the previous estimates. However, given the uncertainty in the parameter estimates, the difference is not statistically significant; the measured values are less than 1.5 standard deviations away from the mean.

We determined the mean values for each parameter distribution to create the mean parameter set (*P*_*mean*_), and simulated the model with these parameter values. This parameter set is given in Table A in S1 Text. The resulting time-course and dose-response curves compared to data are shown in Fig 4B. The mean squared error for the mean parameter set is 6.4 × 10^−4^, which is close to the mean squared error for *P*_*opt*_ (1.3 × 10^−4^).

The correlation between pairs of parameters can be calculated along with the individual distributions. A graphical representation of the correlations among the 8 parameters is given in S2 Fig. Note that *k*_*Ga*_ and *k*_*Gd*_ are still strongly correlated. There are weaker correlations between *k*_*RL*_ and *k*_*RLm*_ and between *k*_*Rs*_ and *k*_*Rd*1_, which represent pairs of rate constants for opposing reactions in ligand binding/unbinding and receptor synthesis/degradation, respectively.

### Model 2: Mechanistic spatial model for yeast cell polarization

To capture the spatiotemporal dynamics of yeast cell polarization during mating, one needs a mechanistic spatial model. In this model, protein spatial dynamics are driven by two processes: surface diffusion on the cell membrane and reactions with other proteins in the system. This leads to a system of reaction-diffusion equations, similar to the model presented in [38]. The first six equations represent the dynamics of the heterotrimeric G-protein cycle, and the remaining equations represent the dynamics of the Cdc42 G-protein cycle. The distance unit is *μ*m, the time unit is seconds, and concentration is measured as the number of molecules per unit surface area or volume (except for the ligand *L*, which is measured in nM).

$$\frac{\partial [R]}{\partial t}=D_{R}\nabla_{m}^{2}\left[R\right]-k_{RL}\left[L\right]\left[R\right]+k_{RLm}\left[RL\right]-k_{Rd0}\left[R\right]+p_{s}k_{Rs}$$ (5)

$$\frac{\partial [RL]}{\partial t}=D_{RL}\nabla_{m}^{2}\left[RL\right]+k_{RL}\left[L\right]\left[R\right]-k_{RLm}\left[RL\right]-k_{Rd1}\left[RL\right]$$ (6)

$$\frac{\partial [G]}{\partial t}=D_{G}\nabla_{m}^{2}\left[G\right]-k_{Ga}\left[RL\right]\left[G\right]+k_{G1}\left[Gd\right]\left[Gbg\right]$$ (7)

$$\frac{\partial [Ga]}{\partial t}=D_{Ga}\nabla_{m}^{2}\left[Ga\right]+k_{Ga}\left[RL\right]\left[G\right]-k_{Gd}\left[Ga\right]$$ (8)

$$\frac{\partial [Gbg]}{\partial t}=D_{Gbg}\nabla_{m}^{2}\left[Gbg\right]+k_{Ga}\left[RL\right]\left[G\right]-k_{G1}\left[Gd\right]\left[Gbg\right]$$ (9)

$$\frac{\partial [Gd]}{\partial t}=D_{Gd}\nabla_{m}^{2}\left[Gd\right]+k_{Gd}\left[Ga\right]-k_{G1}\left[Gd\right]\left[Gbg\right]$$ (10)

$$\begin{array}{ll} \frac{\partial \left[C24m\right]}{\partial t} & =D_{C24m}\nabla_{m}^{2}\left[C24m\right]+k_{24cm0}\left(Gbg_{n}^{*}\right)\left[C24c\right]+k_{24cm1}\left(B1^{*}\right)\left[C24c\right]\\ & \;-k_{24mc}\left[C24m\right]-k_{24d}\left[Cla4a\right]\left[C24m\right] \end{array}$$ (11)

$$\frac{\partial [C42]}{\partial t}=D_{C42}\nabla_{m}^{2}\left[C42\right]-k_{42a}\left[C24m\right]\left[C42\right]+k_{42d}\left[C42a\right]$$ (12)

$$\frac{\partial [C42a]}{\partial t}=D_{C42a}\nabla_{m}^{2}\left[C42a\right]+k_{42a}\left[C24m\right]\left[C42\right]-k_{42d}\left[C42a\right]$$ (13)

$$\frac{\partial [B1m]}{\partial t}=D_{B1m}\nabla_{m}^{2}\left[B1m\right]+k_{B1cm}\left[C42a\right]\left[B1c\right]-k_{B1mc}\left[B1m\right]$$ (14)

$$\frac{\partial [Cla4a]}{\partial t}=k_{Cla4a}\left(C42a_{t}^{*}\right)-k_{Cla4d}\left[Cla4a\right].$$ (15)

The coefficients are given by

$$\begin{array}{cc} & B1^{*}=\frac{B1_{t}^{*}}{1+{\left(\gamma Gbg_{n}^{*}\left[B1m\right]\right)}^{-h}},\;B1_{t}^{*}=\frac{\int_{S}\left[B1m\right]ds}{SA},\;\gamma =\frac{SA}{2\int_{S}\left[B1m\right]ds},\\ & Gbg_{n}^{*}=\frac{1}{1+{\left(\delta \left(Gbg_{n}\right)\right)}^{-q}},\;\delta =\frac{SA}{\int_{S}\left(Gbg_{n}\right)ds},\;\left(Gbg_{n}\right)=\frac{\left[Gbg\right]}{{\left[G\right]}_{0}},\\ & C42a_{t}^{*}=\frac{\int_{S}\left[C42a\right]ds}{SA},\\ & p_{s}=\frac{\left[C42a\right]}{C42a_{t}^{*}}\;\text{if}\;C42a_{t}^{*}>0\text{,}\;\text{else}\;p_{s}=1\text{,} \end{array}$$

where *SA* is the surface area of the cell. The initial conditions are given by

$$\begin{array}{cc} & {\left[R\right]}_{0}=R_{t}/SA\text{,}\;\text{where}\;R_{t}\;\text{is}\;\text{the}\;\text{total}\;\text{amount}\;\text{of}\;R\text{,}\\ & {\left[G\right]}_{0}=G_{t}/SA\text{,}\;\text{where}\;G_{t}\;\text{is}\;\text{the}\;\text{total}\;\text{amount}\;\text{of}\;G\text{,}\\ & {\left[C42\right]}_{0}=C42_{t}/SA\text{,}\;\text{where}\;C42_{t}\;\text{is}\;\text{the}\;\text{total}\;\text{amount}\;\text{of}\;C42\text{,}\\ & {\left[RL\right]}_{0}=0,{\left[Ga\right]}_{0}=0,{\left[C24m\right]}_{0}=0,{\left[C42a\right]}_{0}=0,{\left[B1m\right]}_{0}=0.\\ & \left[Gd\right]={\left[G\right]}_{0}-\left[G\right]-\left[Ga\right],\\ & \left[Gbg\right]={\left[G\right]}_{0}-\left[G\right]. \end{array}$$

The conservation equations are

$$\begin{array}{cc} & V\cdot \left[C24c\right]=C24_{t}-\int_{S}\left[C24m\right]ds,\\ & V\cdot \left[B1c\right]=B1_{t}-\int_{S}\left[B1m\right]ds, \end{array}$$

where *C*24_*t*_ and *B*1_*t*_ are the total amounts of *C*24 and *B*1 respectively, *V* is the volume of the cell, and [*C*24*c*] and [*B*1*c*] are the concentrations of *C*24 and *B*1, respectively, in the cytoplasm. Thus the total amounts of Bem1 and Cdc24 are conserved. Estimates from previous work and ranges for the parameters are given in Table 3.

**Table 3:** Parameter estimates and ranges from previous work. *SA* denotes cell surface area and *V* denotes cell volume. The distance unit is *μ*m, the time unit is seconds, and concentration is measured as the number of molecules per unit surface area or volume (unless otherwise specified).

Parameter	Description	Previous estimate	Range	Ref.
*D*_*R*_	Diffusion of R	0.001	±10%	[60, 61]
*D*_*RL*_	Diffusion of RL	0.001	±10%	[60, 61]
*D*_*G*_	Diffusion of G	0.01	[0.005, 0.02]	[60, 61]
*D*_*Ga*_	Diffusion of Ga	0.01	[0.005, 0.02]	[60, 61]
*D*_*Gbg*_	Diffusion of Gbg	0.01	[0.005, 0.02]	[60, 61]
*D*_*Gd*_	Diffusion of Gd	0.01	[0.005, 0.02]	[60, 61]
*D*_*C*24*m*_	Diffusion of C24m	0.01	[0.005, 0.02]	[60, 61]
*D*_*C*42_	Diffusion of C42	0.01	[0.005, 0.02]	[60, 61]
*D*_*C*42*a*_	Diffusion of C42a	0.01	[0.005, 0.02]	[60, 61]
*D*_*B*1*m*_	Diffusion of B1m	0.01	[0.005, 0.02]	[60, 61]
*k*_*RL*_	RL association	2 × 10^−3^ nM^−1^s^−1^	±10%	[37]
*k*_*RLm*_	RL dissociation	10^−2^	±10%	[37]
*k*_*Rd*0_, *k*_*Rd*1_	R internalization	4 × 10^−4^	±10%	[37]
*k*_*Rs*_	R synthesis	4/*SA*	±10%	[37]
*k*_*Ga*_	G-protein activation	10^−5^ × *SA*	±10%	[37]
*k*_*Gd*_	G-protein deactivation	0.1	±10%	[37]
*k*_*G*1_	Heterotrimer association	1	±10%	[37]
*k*_42*d*_	Cdc42 deactivation	0.02	[0.02, 2]	[60]
*k*_42*a*_	Cdc42 activation	10^−5^ × *SA*	[10^−5^, 10^−3^] × *SA*	[60]
*k*_24*cm*0_	G*βγ* recruitment of Cd24	0.04 × *V*/*SA*	[0.004, 0.4] × *V*/*SA*	[60]
*k*_24*cm*1_	Bem1 recruitment of Cdc24	3.3 × 10^−3^ × *V*	[3.3 × 10^−4^, 3.3 × 10^−2^] × *V*	[60]
*k*_24*mc*_	Cdc24, membrane to cytoplasm	1	[0.1, 1]	[60]
*k*_*B*1*mc*_	Bem1, membrane to cytoplasm	0.01	[0.01, 1]	[60]
*k*_*B*1*cm*_	Bem1, cytoplasm to membrane	10^−5^ × *V*	[10^−5^, 10^−3^] × *V*	[60]
*k*_*Cla*4*a*_	Cla4 activation	0.006	[0.0006, 0.06]	[60]
*k*_*Cla*4*d*_	Cla4 deactivation	0.01	[0.001, 0.1]	[60]
*k*_24*d*_	Negative regulation of Cdc42 cycle	*SA*/3000	[0.1, 10] × *SA*/3000	[60]
*q*	Hill coefficient for $Gng_{n}^{*}$	100	[1, 100]	[38, 60]
*h*	Hill coefficient for *B*1*	8	[1, 8]	[38, 60]
*C*24_*t*_	Total Cdc24	2000	[1000, 3000]	[38]
*B*1_*t*_	Total Bem1	3000	[2000, 5000]	[38]
*R*_*t*_	Total receptor	10000	±10%	[38]
*G*_*t*_	Total G-protein	10000	±10%	[37]
*C*42_*t*_	Total Cdc42	10000	[5000, 20000]	[38]

In our numerical simulations, the cell membrane is simulated as a circle centered at the origin with radius 2 *μ*m. The pheromone input is administered as a gradient from the positive *x*-direction with midpoint of 10 nM and slope of 0.1 nM/*μ*m. The surface diffusion of a quantity *W* on a circle is given by

$$\begin{array}{c} \nabla_{m}^{2}W=W_{ss} \end{array}$$

where *s* is an arc length parameter, *ds*^2^ = *dx*^2^ + *dy*^2^. The computational domain is parametrized by *α* ∈ [0, 2*π*], where *α* denotes the angle from the negative *x*-axis. The numerical method utilizes a second order finite difference discretization for the spatial derivatives and an implicit Crank-Nicolson method for the time derivative. The spatial mesh consists of 400 equally spaced points. Each simulation is run to steady state (*t* = 1, 000s). More detail about the numerical method can be found in the Supplementary Material (S2 Text).

#### Surrogate model construction using an underdetermined fit

The quantity of interest in this model is the extent of cell polarization, more specifically, the extent of active Cdc42 polarization. Therefore, we consider a scalar function of active Cdc42 (*C*42*a*), which we call the polarization factor (*PF*) of active Cdc42, defined by

$$\begin{array}{c} PF\left(C42a\right)=1-2\frac{S_{p}\left(C42a\right)}{SA}, \end{array}$$

where *S*_*p*_(*C*42*a*) is the surface area at the front of the cell that encompasses half of the polarized component C42a [60]. An unpolarized cell would have a *PF* of 0 and an infinitely polarized cell would have a *PF* of 1. One disadvantage of this measure of polarization is that it considers only the shape of the *C*42*a* curve and not the magnitude; thus *PF* may be close to 1 even if only a small amount of Cdc42 is activated and localized. To remedy this issue, we introduce a cut-off function:

$$\begin{array}{c} z=PF\left(C42a\right)\times \frac{{\left(ax\right)}^{n}}{1+{\left(ax\right)}^{n}} \end{array}$$ (21)

where $a=\frac{2*SA}{C42_{t}}$ and *x* = max(*C*42*a*). This cut-off function penalizes the cases in which the maximum amount of *C*42*a* is less than half of the initial value of *C*42. We choose the exponent to be *n* = 5 to produce a reasonably sharp cut-off.

We perform polynomial fitting using a Legendre polynomial basis to fit the response function *PF*(*C*42*a*). The parameters *q* and *h* are mapped to [−1, 1] on a linear scale and all other parameters are mapped to [−1, 1] on a log scale. We use a 5th order polynomial as our surrogate model. Recall that to fit a 5th degree polynomial for the full 35-dimensional parameter space, we require at least $\left(\begin{array}{c} 40\\ 35 \end{array}\right)=658,008$ sample points for interpolation or least squares fitting. Since each sample point involves simulating a large PDE model, we wish to decrease the number of sample points to save computation time. However, decreasing the number of sample points results in an underdetermined system. One technique for solving such a system is compressed sensing—in particular, *ℓ*_1_-minimization. We used *ℓ*_1_-MAGIC, a collection of MATLAB subroutines by Emmanuel Candes, to perform *ℓ*_1_-minimization with quadratic constraints [62].

5, 000 points are used to fit a 5th order polynomial in the full 35-dimensional parameter space. The accuracy of the polynomial is evaluated on an additional 500 uniformly random points. A histogram of the errors between the model and polynomial is shown in S3 Fig. The mean absolute error is 0.12 with most errors between -0.2 and 0.2.

#### Parameter sensitivity analysis helps to reduce number of free parameters

Once we have established a polynomial surrogate model, we can analytically compute parameter sensitivities. Assuming that each parameter is uniformly distributed in [−1, 1], the sensitivity of the response function *z* to *j*-th parameter is $S_{j}=(\frac{1}{2}{)}^{35}\int_{{\left[-1,1\right]}^{35}}\frac{\partial z}{\partial y_{j}}dy$. Note that since the parameters are all mapped to the same range, there is no inherent bias due to differing magnitudes of parameter values. The parameter sensitivity coefficients for the full 35-parameter model are given in S4 Table.

We observe that many of the parameters have small sensitivity coefficients, and the parameters of primary importance are those associated with the Cdc42 cycle dynamics. Based on the parameter sensitivities in S4 Table, we can reduce the parameter count by eliminating the parameters to which polarization is least sensitive. If we consider only the parameters whose sensitivity coefficients are greater than 0.01, we are left with only 15 parameters: *B*1_*t*_, *k*_24*d*_, *C*24_*t*_, *k*_*Cla*4*a*_, *k*_*Cla*4*d*_, *k*_*B*1*cm*_, *k*_24*cm*0_, *k*_*B*1*mc*_, *D*_*c*42*a*_, *h*, *D*_*c*42_, *q*, *k*_42*d*_, *k*_24*cm*1_, and *k*_42*a*_.

In this 15-dimensional subspace, we can again perform polynomial fitting to obtain a surrogate model. We use 6000 points to fit a 5th order polynomial using *ℓ*1-minimization. We perform 12-fold cross validation to analyze the error in the polynomial fit, and find that the mean absolute error is 0.14. A histogram of the error in the polynomial approximation is given in S4 Fig. Using this reduced model, we may again compute the parameter sensitivities. The resulting sensitivities are given in Table 4, and are largely consistent with the parameter sensitivities from the full 35-parameter model. Surprisingly, the diffusion constants *D*_*c*42*a*_ and *D*_*c*42_ have high sensitivities along with the parameters directly involved in the Bem1-Cdc24-Cdc42 positive feedback loop activating Cdc42.

**Table 4:** Sensitivity coefficients, in order of ascending magnitude, for the reduced 15-parameter PDE model based on a 5th order polynomial fit using 6,000 sample points.

Parameter	Sensitivity
*k*_24*d*_	1.4 × 10^−3^
*k*_*Cla*4*a*_	−4.0 × 10^−3^
*k*_24*cm*0_	−1.3 × 10^−2^
*B*1_*t*_	1.7 × 10^−2^
*k*_*Cla*4*d*_	2.5 × 10^−2^
*C*24_*t*_	4.4 × 10^−2^
*k*_*B*1*cm*_	5.5 × 10^−2^
*k*_*B*1*mc*_	−5.6 × 10^−2^
*D*_*c*42*a*_	−5.9 × 10^−2^
*q*	6.1 × 10^−2^
*D*_*c*42_	7.0 × 10^−2^
*h*	7.7 × 10^−2^
*k*_24*cm*1_	9.8 × 10^−2^
*k*_42*d*_	−1.3 × 10^−1^
*k*_42*a*_	1.4 × 10^−1^

#### Parameter estimation using yeast cell polarization data

We wished to estimate the model parameters that could produce polarization by fitting to experimental data. The key species in yeast polarization is active Cdc42 (C42a) which we can monitor using the reporter Ste20-GFP, a fusion protein that binds active Cdc42 and possesses a fluorescent tag [54]. We treat yeast cells containing Ste20-GFP with 10 nM of *α*-factor for 60 min to correspond to the steady-state polarization in the model. We assess the spatial distribution of Ste20-GFP by confocal microscopy of fixed cells, followed by quantitative image processing (see Methods). The Ste20-GFP polarization profile is converted into a polarization factor to be compared with the model simulations.

**Fig 5:**
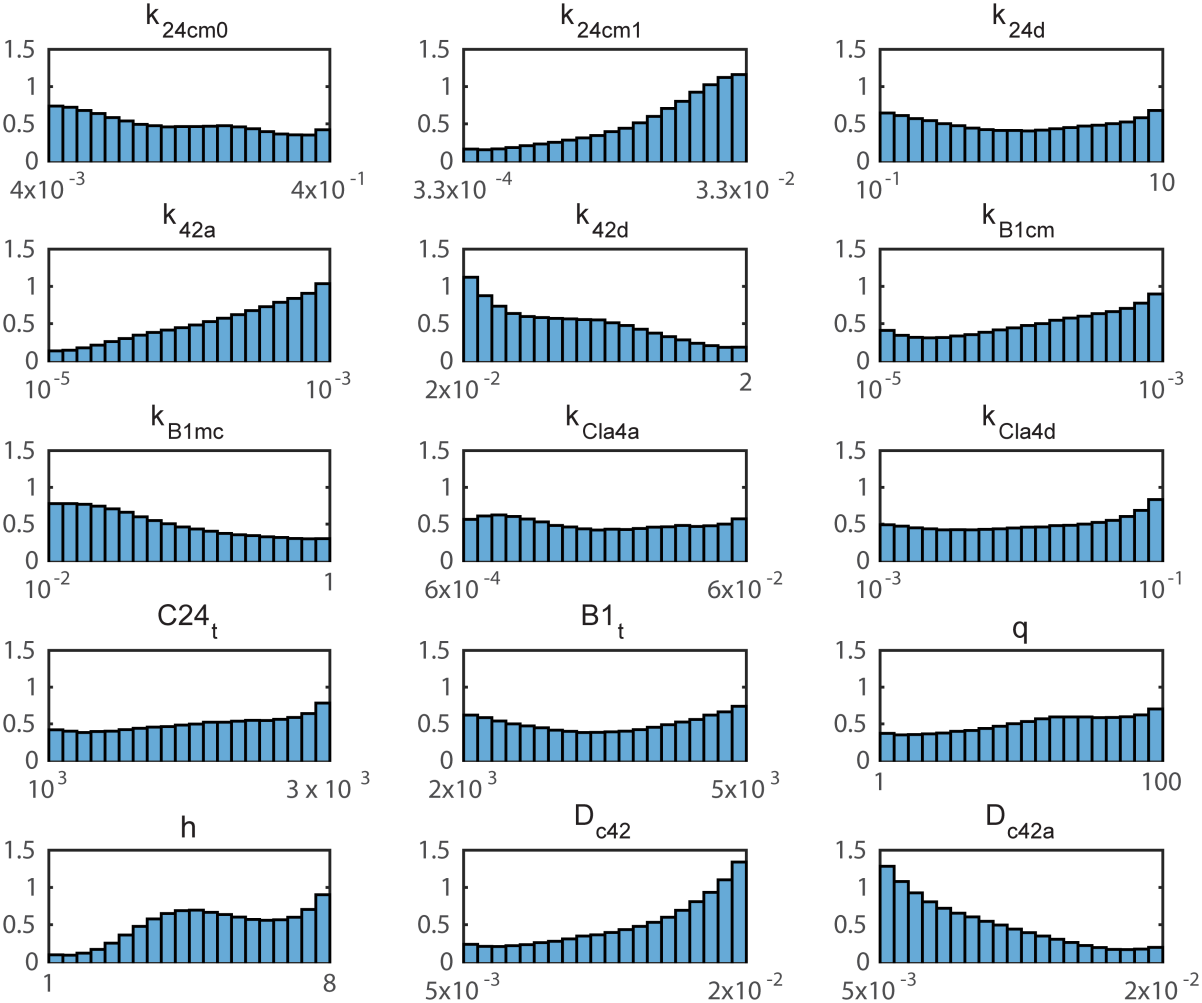
MCMC results for PDE model. Parameter distributions based on MCMC with chain length 2 × 10^6^ for the reduced 15-parameter PDE model. The parameter range is a log-scale except for the parameters *q* and *h* which span a linear scale.

With these data, we can perform parameter estimation using the 15-parameter polynomial surrogate model and an MCMC method. Fig 5 shows the parameter distributions obtained via MCMC with Markov chain length 2 × 10^6^. As done in the ODE model, the correlation coefficients between parameters can be calculated and they are displayed graphically in S5 Fig. There are a few key observations. First, there is significant uncertainty in many of the parameters (particularly those with small sensitivity coefficients). This implies that, given the current data, we are not able to infer much information about the parameters. The broad distributions also suggest that a wide range of parameter values are compatible with polarization in the model. Second, we see that several of the parameters tend toward the boundary (e.g. *k*_42*a*_, *k*_42*d*_, *D*_*c*42_, *D*_*c*42*a*_), indicating that the parameter ranges should be extended. In particular, a faster diffusion constant for inactive Cdc42 (*D*_*c*42_) and a slower diffusion constant for active Cdc42 (*D*_*c*42*a*_) promote polarization. Lastly, we see that the parameters are not strongly correlated, with the exception of some correlation between *k*_42*a*_ and *k*_42*d*_ and between *h* and *q*.

#### Identifying an optimal parameter set for the PDE model

Sometimes it is desirable to obtain a single best parameter estimate (e.g. maximum likelihood) to visualize how closely the model can fit the data, and to determine the parameter values at that best fit. In this example, due to the large amount of uncertainty in the parameter distributions, there is no clear choice for such a point estimate. In fact, given the limited data relative to the large number of parameters, multiple “best” parameter estimates may exist. One approach is to take the mean of the MCMC iterates (*P*_*mean*_) as was done with the ODE model in the previous section. This parameter set and its corresponding polarization factor are given in Table B in S1 Text. However, the large degree of parametric uncertainty suggests this estimate may not produce a good fit.

**Fig 6:**
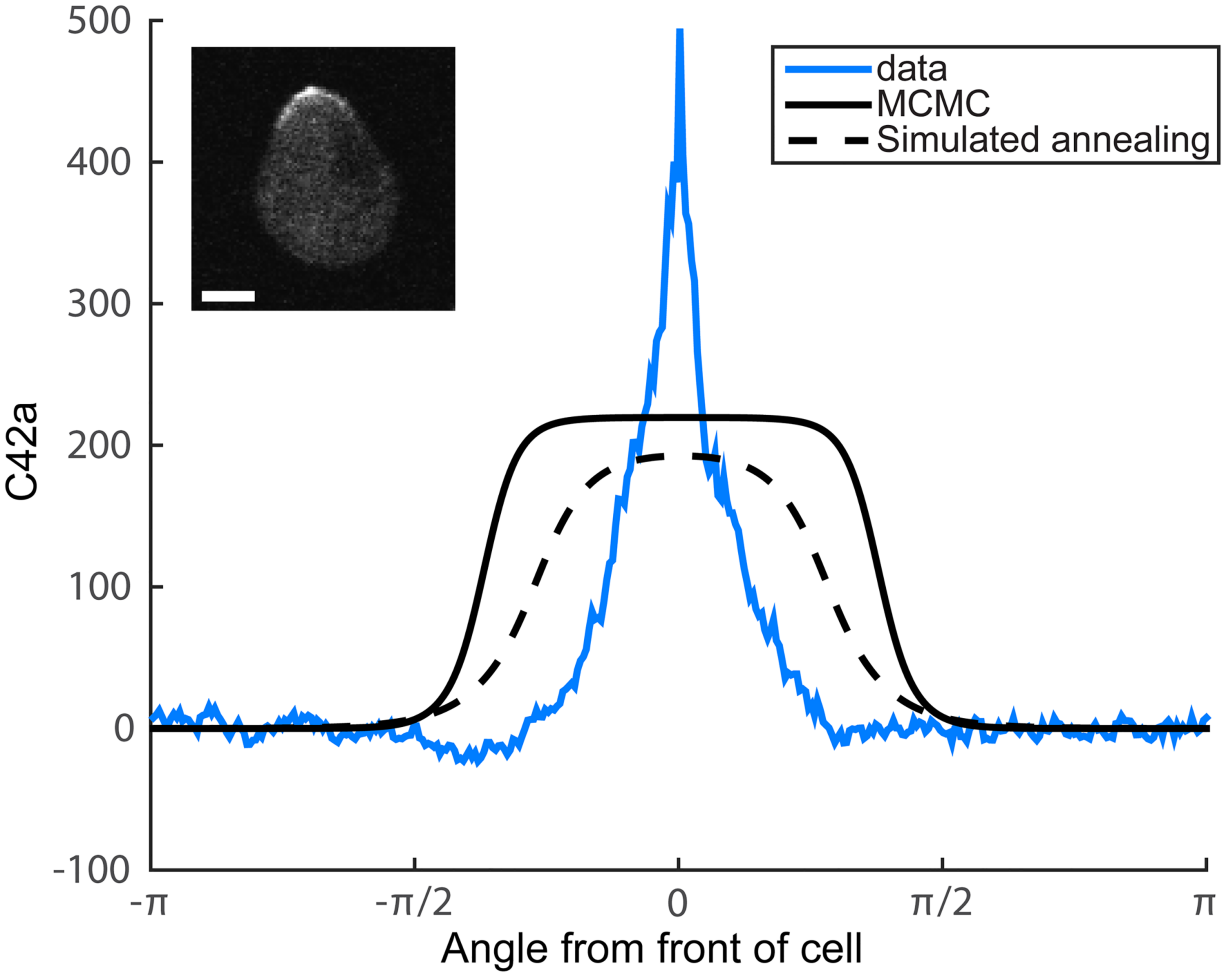
MCMC vs. optimization results. Steady state solutions for the mean parameter set from the MCMC (solid black) and a parameter set identified via simulated annealing (dashed black). Polarization is depicted by the concentration of active Cdc42 (C42a, number of molecules/*μ*m^2^) over the angular range [−*π*, *π*]. The mean polarization of the experimental data (*n* = 20 cells) is shown in blue in arbitrary units. A sample cell, treated with 10 nM *α*-factor for 60 min, shows the membrane polarization profile of Ste20-GFP, a reporter for active Cdc42 (upper left). Scale bar = 2 *μ*m.

Another option is to use an optimization method such as simulated annealing to improve upon *P*_*mean*_ which is used as the starting point. Note that the polynomial surrogate model greatly speeds up this optimization. Fig 6 shows the steady state solutions for both the MCMC mean and a parameter set identified via simulated annealing (SA). Indeed, the SA gives a better fit to the data (*PF*_*SA*_ = 0.64, *PF*_*mean*_ = 0.57, *PF*_*data*_ = 0.87; see Supplement for parameter sets). However, the broadness of the model simulations compared to the experimental data suggests that the model structure is unable to capture the sharp polarization observed in the cell. One possible explanation is unmodeled dynamics. Nevertheless, the Bayesian parameter estimation provides valuable information about the range of parameters in the model that allow polarization, the most likely parameter values, and the uncertainty in the parameters given the current data.

## Discussion

In this work we apply novel methods from uncertainty quantification to perform parameter sensitivity analysis and parameter estimation of two models of yeast mating. The central innovation is the construction of polynomial surrogate models to replace simulation for calculating the model output. We demonstrate the accuracy of the polynomials by cross-validation on random sample points left out from the polynomial fitting. For Bayesian parameter estimation, the method provides a dramatic reduction in computational cost.

### Computational speed-up

Typically, MCMC requires a model evaluation at every iteration. Since our Markov chain length for the 15-parameter model was 2 × 10^6^, we would require 2 × 10^6^ evaluations of the PDE model to steady state. It would likely take even more iterations for the MCMC to converge for the full 35-parameter model. The PDE is solved with an implicit method implemented in Fortran, and each evaluation takes 40-60 minutes of CPU time. Thus, the full MCMC would require at least ∼200 years of CPU time. Further, MCMC is not inherently parallelizable, although advancements have been made in parallel MCMC methods [63–66].

Using the polynomial surrogate, we are able to practically eliminate the cost of MCMC by evaluating only a polynomial at each MCMC iteration. Computing a chain of length of 2 × 10^6^ takes only a few hours in MATLAB. In place of this cost, we must evaluate the full PDE model at the sample points used to fit the polynomial. For our full 35-parameter model, we use 5000 sample points to fit a polynomial to perform the sensitivity analysis. We then are able to reduce the parameter count to 15, and use 6000 additional samples to fit a polynomial in the reduced parameter space. Thus we require 11,000 model evaluations in total. There is also some cost to fit the polynomial via *ℓ*_1_-minimization, which is of the order of several hours. The time required to evaluate the polynomial is considered to be negligible compared to the time required to solve the PDE. Thus we have a roughly 180-fold reduction in computational cost compared to the MCMC without a polynomial surrogate. In addition, the samples are independent so that the model evaluations to produce these samples can easily be computed in parallel.

The computational savings in the ODE test model are not as dramatic, since the ODE model is inexpensive to solve. In numerical tests for the 2-parameter ODE model with a 10th degree polynomial surrogate, we found a 20% reduction in CPU time in evaluating the polynomial vs. evaluating the model directly. In the 8-parameter model with a 5th degree polynomial surrogate, we found a more than 10-fold reduction in CPU time; we believe the greater reduction in cost is afforded by the lower polynomial degree. The computational savings afforded by using polynomial surrogates will vary depending on the ODE solver, the degree of the polynomials, and the time step required to solve the ODE. Whether a problem warrants the use of surrogate models will generally depend on the cost of evaluating the original model, the number of sample (data) points required for accurate parameter estimation, and the polynomial degree required to fit the model output.

### Possible limitations of uncertainty quantification methodology

The primary challenge with the method is constructing accurate polynomials. As we demonstrate in the ODE example, more sample points and a higher degree polynomial produce greater accuracy. One concern is the ability of the surrogate polynomials to describe highly nonlinear relationships between parameters and outputs arising from bifurcations. If the model output is discontinuous with respect to the parameters, for example, then the model output will not be well-approximated by polynomials. This issue may exist in the PDE model presented here, since it has previously been shown that the model for some parameter values possesses multistability contributing to the polarization [60]; thus the steady state behavior is discontinuous with respect to the initial conditions. The 5th degree polynomial surrogate produces an error of 0.1 to 0.2. It is likely we can reduce the error by employing more sample points or by using a higher degree polynomial. Alternatively, one can take advantage of Design of Experiments methods [67] to pick more informative sample points to decrease the error. However, we will still not be able to capture the discontinuous nature of the model output.

Another issue is that one may make false assumptions in determining a response function. In the PDE model we choose a response function that quantifies the cell polarization at steady state, and thus we are assuming that the system settles to a steady state. While this seems to be a reasonable assumption for the system presented here, this may not always be the case. If a system has periodic solutions rather than a stable steady state in some region of the parameter space, then one would need to carefully consider how to build an appropriate response function. Unfortunately, it is not always clear a priori whether such solutions exist for a given system.

Finally, a third issue is the combinatorial increase in the number of polynomial coefficients as the number of parameters increases. The 5th degree polynomial for the 35 parameter model possesses 658,008 coefficients and a 100 parameter 5th degree polynomial would possess over 75 million coefficients. For the PDE model we employ compressed sensing methods (*ℓ*_1_-minimization) that allow undersampling to fit higher dimensional polynomials from larger models with fewer sample points. It is possible to adopt advanced sparse regression methods such as *ℓ*_1_-*ℓ*_2_ minimization [68] to further reduce the number of required model evaluations. A second approach is explore optimal sample set design such as the optimal sample selection strategy [45] that, for any given number of samples (model evaluations), finds the parameter sample points to provide a polynomial surrogate nearly as accurate as the one obtained by a much larger number of model evaluations.

### Parameter analysis of yeast mating models yields biological insights

In the yeast G-protein ODE model, the parameter distributions inferred from the time-course and dose-response data are consistent with the parameter estimates and experimental measurements from [37]. For example, the peaks for *k*_*Ga*_ and *k*_*Gd*_ are very close to the previous maximum likelihood estimates. Interestingly, the parameter estimates for *k*_*RL*_ and *k*_*RLm*_ are close to the measured values found in [37], but they are at least one to two orders of magnitude larger than the estimated values from three other groups [69–71]. One possible explanation is the use of fluorescent analogs of *α*-factor in some of the earlier work, but this discrepancy needs to be addressed in future work.

The PDE model shows broad distributions for nearly all 15 parameters examined indicating that a wide range of parameter values are compatible with good polarization of active Cdc42. The fact that the feasible region of the parameter *q*, representing the cooperativity of the interaction between G*βγ* and Cdc24, spans the full range from 1 to 100 demonstrates that the high value (*q* = 100) previously used in the model [38] is not necessary for polarization, and that lower values (e.g. *q* = 1 to 10) are almost equally probable. These lower cooperativity values corresponding to smaller Hill exponents are more plausible from a mechanistic standpoint. In addition, several parameters (*k*_24*cm*1_, *k*_42*a*_, *k*_42*d*_, *D*_*c*42_, and *D*_*c*42*a*_) show peaks at one or the other side of the distribution indicating that the previous estimates may miss the most likely parameter range. The diffusion constants *D*_*c*42_ and *D*_*c*42*a*_ were assigned the same value in our previous model [38, 61], but in this work *D*_*c*42_ shows a preference for higher values, whereas *D*_*c*42*a*_ shows a preference for lower values. Recent measurements by Bendezú et al. [72] in the fission yeast *S. pombe* found that inactive Cdc42 had a 10-fold faster diffusion rate than active Cdc42 consistent with the trends in our parameter distributions.

This work also highlights the inability of the current PDE model to produce the sharp polarization peak of active Cdc42 observed in cells. One explanation is that the model is missing important dynamics or positive feedback mechanisms that enhance cell polarization. In the future, we plan to include additional spatial dynamics such as the polarized transport of Cdc42 to the front of the projection, which is absent from the model.

The broadness of the obtained parameter distributions also implies that the current data are insufficient to obtain tight parameter estimates. In this study we focused on identifying parameter values that would produce polarization in the model versus an unpolarized state. Further data can be collected tracking the spatial dynamics of the other species in the model such as G*βγ*, Cdc24, and Bem1 in both wild-type and mutant yeast strains. The additional data along with model modifications should result in narrower parameter distributions and a better fit to the total system dynamics.

### Application of polynomial surrogates to other model analysis methods

In our analysis, we presented only the sensitivity measure $S_{j}=E(\frac{\partial z}{\partial p_{j}})$. The advantage of this sensitivity measure is its simplicity; it is easy to compute analytically when *z* is a known function and it usually provides a good measure of sensitivity when the relationship between *z* and *p*_*j*_ is monotonic. However, if the relationship between *z* and *p*_*j*_ is non-monotonic or highly nonlinear, *S*_*j*_ may not be a desirable measure. In these cases, other measures of sensitivity may be a better choice such as variance-based sensitivity measures or the partial rank correlation coefficient [8, 73]. The use of polynomial chaos expansions to approximate variance-based sensitivities has been explored previously in [31]. Other derivative-based sensitivity measures have also been proposed [74], which can be computed analytically using the polynomial chaos expansion.

Polynomial surrogates may also be used in methods for parameter estimation not addressed in this paper. In principle, polynomial surrogates can be applied to any type of model for which parameter ranges are known, and for any sampling-based method that requires model evaluations. By fitting polynomials to the quantities for which data is available, every model evaluation in a computational method can be replaced by a polynomial evaluation. While we have demonstrated this here only in the context of a Markov chain Monte Carlo method, the same principles may be used to accelerate the computations involved in other Bayesian methods for parameter estimation, such as rejection sampling and sequential Monte Carlo.

Yet another potential application of polynomial surrogates is to accelerate methods for Bayesian model selection. The idea behind Bayesian model selection is that we can recover a probability distribution for a model index parameter *m* enumerating different models, providing information on the likelihoods of the candidate models given the available data. In essence this is still a parameter estimation problem, and established methods for parameter estimation can be adapted for model selection. Polynomial surrogates can be used to accelerate these methods which include Bayesian rejection sampling, sequential Monte Carlo, population annealing, and MCMC [20, 52, 53, 75, 76]. Model selection is of great importance in systems biology since uncertainty in the model structure may significantly impact the conclusions of parameter inference [39].

## Supporting information

S1 Fig Schematic reaction diagram of the yeast mating signal transduction pathway. Arrows indicate the conversion of protein species from inactive to active form or from cytoplasmic localization to membrane localization (where the protein is active). Solid dots represent reactions catalyzed by the connected proteins. Lines terminating in a vertical bar (instead of an arrow) represent inhibition. Species and reactions are described in the main text.
(EPS)

S2 Fig Model 1 parameter correlations. Correlations between the 8 kinetic parameters in the MCMC chain using the polynomial surrogate of Model 1.
(EPS)

S3 Fig Model 2 35-dimensional polynomial error. Error in the 35-dimensional polynomial surrogate function for Model 2 fit using 5000 points, and measured (tested) at 500 uniform random samples.
(EPS)

S4 Fig Model 2 15-dimensional polynomial error. Error in the 15-dimensional polynomial surrogate function for Model 2 fit using 6000 points, and measured (tested) via 10-fold cross-validation.
(EPS)

S5 Fig Model 2 parameter correlations. Correlations between the parameters in the MCMC chain using the 15-dimensional polynomial surrogate of Model 2.
(EPS)

S1 Table Parameter values for model 1. Parameter values are taken from [37]. Values for *k*_*Ga*_ and *k*_*Gd*_ were estimated based on least-squares fit to time course and dose-response data.
(PDF)

S2 Table Experimental data. Experimental data for the given time points and *α*-factor levels from [37], and the resulting data. Output is the fraction of free G*βγ* (*Gbg*/*Gt*). Data are given as mean ± standard deviation.
(PDF)

S3 Table Parameter ranges for model 1. Ranges for the kinetic parameters used for parameter estimation of all 8 parameters in Model 1 (heterotrimeric G-protein model).
(PDF)

S4 Table Sensitivity coefficients for model 2. Sensitivity coefficients, in order of ascending magnitude, from sensitivity analysis of all 35 parameters in Model 2 using a 5th order surrogate polynomial fit to 5000 sample points.
(PDF)

S1 Text Parameter sets. (PDF)

S2 Text Numerical methods. (PDF)

## References

[1] Edelstein-Keshet L. Mathematical models in biology. SIAM; 2005.

[2] Chou CS, Friedman A. Introduction to Mathematical Biology: Modeling, Analysis, and Simulations. Springer; 2016.

[3] Gershenfeld NA. The Nature of Mathematical Modeling. Cambridge University Press; 1999.

[4] Slepchenko BM, Schaff JC, Carson JH, Loew LM. Computational cell biology: spatiotemporal simulation of cellular events. Annu Rev Biophys Biomol Struct. 2002;31:423–441. doi: 10.1146/annurev.biophys.31.101101.140930 11988477 10.1146/annurev.biophys.31.101101.140930

[5] Fall CP, Marland ES, Wagner JM, Tyson JJ. Computational Cell Biology. Springer Science & Business Media; 2007.

[6] Ljung L. System Identification In: Signal Analysis and Prediction. Applied and Numerical Harmonic Analysis. Birkhäuser, Boston, MA; 1998 p. 163–173.

[7] Bard Y. Nonlinear parameter estimation. Academic Press; 1974.

[8] Marino S, Hogue IB, Ray CJ, Kirschner DE. A methodology for performing global uncertainty and sensitivity analysis in systems biology. Journal of Theoretical Biology. 2008;254:178–196. doi: 10.1016/j.jtbi.2008.04.011 18572196 10.1016/j.jtbi.2008.04.011 PMC2570191

[9] Banks HT, Bortz DM. A parameter sensitivity methodology in the context of HIV delay equation models. Journal of Mathematical Biology. 2005;50:607–625. doi: 10.1007/s00285-004-0299-x 15614552 10.1007/s00285-004-0299-x

[10] Cariboni J, Gatelli D, Liska R, Saltelli A. The role of sensitivity analysis in ecological modelling. Ecological Modelling. 2007;203:167–182. doi: 10.1016/j.ecolmodel.2005.10.045

[11] Dancik GM, Jones DE, Dorman KS. Parameter estimation and sensitivity analysis in an agent-based model of *Leishmania major* infection. Journal of Theoretical Biology. 2010;262:398–412. doi: 10.1016/j.jtbi.2009.10.007 19837088 10.1016/j.jtbi.2009.10.007 PMC2789658

[12] Blower SM, Dowlatabadi H. Sensitivity and uncertainty analysis of complex models of disease transmission: an HIV model, as an example. International Statistical Review. 1994;62(2):229–243. doi: 10.2307/1403510

[13] Cho KH, Shin SY, Kolch W, Wolkenhauer O. Experimental design in systems biology, based on parameter sensitivity analysis using a Monte Carlo method: A case study for the TNF*α*-mediated NF-*κ*B signal transduction pathway. Simulation. 2003;79(12):726–739. doi: 10.1177/0037549703040943

[14] Mahdavi A, Davey RE, Bhola P, Yin T, Zandstra PW. Sensitivity analysis of intracellular signaling pathway kinetics predicts targets for stem cell fate control. PLoS Computational Biology. 2007;3(7):e130 doi: 10.1371/journal.pcbi.0030130 17616983 10.1371/journal.pcbi.0030130 PMC1913098

[15] Chu Y, Jayaraman A, Hahn J. Parameter sensitivity analysis of IL-6 signalling pathways. IET Systems Biology. 2007;1(6):342–352. doi: 10.1049/iet-syb:20060053 18203580 10.1049/iet-syb:20060053

[16] Pruett W, Hester R. The Creation of Surrogate Models for Fast Estimation of Complex Model Outcomes. PLoS ONE. 2016;11(6):e0156574 doi: 10.1371/journal.pone.0156574 27258010 10.1371/journal.pone.0156574 PMC4892541

[17] Krishna NA, Pennington HM, Coppola CD, Eisenberg MC, Shugart RC. Connecting local and global sensitivities in a mathematical model for wound healing. Bulletin of Mathematical Biology. 2015;77:2294–2324. doi: 10.1007/s11538-015-0123-3 26597096 10.1007/s11538-015-0123-3

[18] Gelman A, Carlin JB, Stern HS, Dunson DB, Vehtari A, others. Bayesian data analysis. 3rd ed CRC Press; 2014.

[19] Xue N, Pan W, Guo Y. Parameter estimation for gene regulatory networks: a two-stage MCMC Bayesian approach. In: 38th Annual International Conference of the Engineering in Medicine and Biology Society (EMBC). IEEE; 2016. 10.1109/EMBC.2016.7590988 28268605

[20] Toni T, Welch D, Strelkowa N, Ipsen A, Stumpf MPH. Approximate Bayesian computation scheme for parameter inference and model selection in dynamical systems. Journal of the Royal Society Interface. 2009;6:187–202. doi: 10.1098/rsif.2008.0172 10.1098/rsif.2008.0172 PMC2658655 19205079

[21] Ghasemi O, Lindsey ML, Yang T, Nguyen N, Huang Y, Jin YF. Bayesian parameter estimation for nonlinear modelling of biological pathways. BMC Systems Biology. 2011;5(Suppl 3):S9 doi: 10.1186/1752-0509-5-S3-S9 22784628 10.1186/1752-0509-5-S3-S9 PMC3287577

[22] Ashyraliyev M, Fomekong-Nanfack Y, Kaandorp JA, Blom JG. Systems biology: parameter estimation for biochemical models. FEBS Journal. 2009;276(4):886–902. doi: 10.1111/j.1742-4658.2008.06844.x 19215296 10.1111/j.1742-4658.2008.06844.x

[23] Moles CG, Mendes P, Banga JR. Parameter estimation in biochemical pathways: a comparison of global optimization methods. Genome Research. 2003;13:2467–2474. doi: 10.1101/gr.1262503 14559783 10.1101/gr.1262503 PMC403766

[24] Ashyraliyev M, Jaeger J, Blom JG. Parameter estimation and determinability analysis applied to Drosophila gap gene circuits. BMC Systems Biology. 2008;2(83). doi: 10.1186/1752-0509-2-83 18817540 10.1186/1752-0509-2-83 PMC2586632

[25] Lillacci G, Khammash M. Parameter estimation and model selection in computational biology. PLoS Computational Biology. 2010;6(3):e1000696 doi: 10.1371/journal.pcbi.1000696 20221262 10.1371/journal.pcbi.1000696 PMC2832681

[26] Xiu D, Karniadakis G. The Weiner-Askey polynomial chaos for stochastic differential equations. SIAM Journal on Scientific Computing. 2002;24(2):619–644. doi: 10.1137/S1064827501387826

[27] Najm HN. Uncertainty Quantification and Polynomial Chaos Techniques in Computational Fluid Dynamics. Annu Rev Fluid Mech. 2008;41(1):35–52. doi: 10.1146/annurev.fluid.010908.165248

[28] Iglesias M, Stuart AM. Inverse problems and uncertainty quantification. SIAM News. 2014; p. 2–3.

[29] Xiu D. Fast Numerical Methods for Stochastic Computations: A Review. Communications in Computational Physics. 2009;5(2-4):242–272.

[30] Xiu D. Efficient collocational approach for parametric uncertainty analysis. Communications in Computational Physics. 2007;2(2):293–309.

[31] Sudret B. Global sensitivity analysis using polynomial chaos expansions. Reliability Engineering & System Safety. 2008;93(7):964–979. doi: 10.1016/j.ress.2007.04.002

[32] Butler MT, Wallingford JB. Planar cell polarity in development and disease. Nat Rev Mol Cell Biol. 2017;18(6):375–388. doi: 10.1038/nrm.2017.11 28293032 10.1038/nrm.2017.11 PMC5826606

[33] Martin-Belmonte F, Perez-Moreno M. Epithelial cell polarity, stem cells and cancer. Nat Rev Cancer. 2011;12(1):23–38. doi: 10.1038/nrc3169 22169974 10.1038/nrc3169

[34] Lee M, Vasioukhin V. Cell polarity and cancer-cell and tissue polarity as a non-canonical tumor suppressor. Journal of Cell Science. 2008;121(8):1141–1150. doi: 10.1242/jcs.016634 18388309 10.1242/jcs.016634

[35] Martin SG, Arkowitz RA. Cell polarization in budding and fission yeasts. FEMS Microbiol Rev. 2014;38(2):228–253. doi: 10.1111/1574-6976.12055 24354645 10.1111/1574-6976.12055

[36] Park HO, Bi E. Central roles of small GTPases in the development of cell polarity in yeast and beyond. Microbiology and Molecular Biology Reviews. 2007;71(1):48–96. doi: 10.1128/MMBR.00028-06 17347519 10.1128/MMBR.00028-06 PMC1847380

[37] Yi TM, Kitano H, Simon MI. A quantitative characterization of the yeast heterotrimeric G protein cycle. Proc Natl Acad Sci USA. 2003;100(19):10764–10769. doi: 10.1073/pnas.1834247100 12960402 10.1073/pnas.1834247100 PMC196877

[38] Yi TM, Chen S, Chou CS, Nie Q. Modeling Yeast Cell Polarization Induced by Pheromone Gradients. Journal of Statistical Physics. 2007;128(1):193–207. doi: 10.1007/s10955-007-9285-1

[39] Babtie AC, Kirk P, Strumpf MPH. Topological sensitivity analysis for systems biology. PNAS. 2014;111(52):18507–18512. doi: 10.1073/pnas.1414026112 25512544 10.1073/pnas.1414026112 PMC4284538

[40] Äijö T, Bonneau R. Biophysically motivated regulatory network inference: progress and prospects. Human Heredity. 2016;81:62–77. doi: 10.1159/000446614 28076866 10.1159/000446614

[41] Farhangmehr F, Maurya MR, Tartakovsky DM, Subramaniam S. Information theoretic approach to complex biological network reconstruction: application to cytokine release in RAW 264.7 macrophages. BMC Systems Biology. 2014;8(77). doi: 10.1186/1752-0509-8-77 24964861 10.1186/1752-0509-8-77 PMC4094931

[42] Candes EJ, Romberg J, Tao T. Robust uncertainty principles: exact signal reconstruction from highly incomplete frequency information. IEEE Trans Inf Theory. 2006;52(2):489–509. doi: 10.1109/TIT.2005.862083

[43] Doostan A, Owhadi H. A non-adapted sparse approximation of PDEs with stochastic inputs. J Comput Phys. 2011;230(8):3015–3034. doi: 10.1016/j.jcp.2011.01.002

[44] Yan L, Guo L, Xiu D. Stochastic collocation algorithms using *ℓ*_1_-minimization. Int J UQ. 2012;2(3):279–293.

[45] Shin Y, Xiu D. Nonadaptive quasi-optimal points selection for least squares linear regression. SIAM Journal on Scientific Computing. 2016;38(1):A385–A411. doi: 10.1137/15M1015868

[46] Brooks SP. Markov chain Monte Carlo method and its application. The Statistician. 1998;47(1):69–100.

[47] Wilkinson DJ. Bayesian methods in bioinformatics and computational systems biology. Briefings in Bioinformatics. 2007;8(2):109–116. doi: 10.1093/bib/bbm007 17430978 10.1093/bib/bbm007

[48] Golightly A, Wilkinson DJ. Bayesian parameter inference for stochastic biochemical network models using particle Markov chain Monte Carlo. Interface Focus. 2011;1(6):807–820. doi: 10.1098/rsfs.2011.0047 23226583 10.1098/rsfs.2011.0047 PMC3262293

[49] Marzouk Y, Xiu D. A stochastic collocation approach to Bayesian inference in inverse problems. Communications in Computational Physics. 2009;6(4):826–847. doi: 10.4208/cicp.2009.v6.p826

[50] Cowles M, Carlin B. Markov chain Monte Carlo convergence diagnostics: A comparative review. Journal of the American Statistical Association. 1996;91(434):883–904. doi: 10.1080/01621459.1996.10476956

[51] Gelman A, Rubin D. Inference from iterative simulation using multiple sequences. Statistical Science. 1992;7(4):457–472. doi: 10.1214/ss/1177011136

[52] Liepe J, Kirk P, Filippi S, Toni T, Barnes CP, Stumpf MPH. A framework for parameter estimation and model selection from experimental data in systems biology using approximate Bayesian computation. Nature Protocols. 2014;9(2):439–456. doi: 10.1038/nprot.2014.025 24457334 10.1038/nprot.2014.025 PMC5081097

[53] Murakami Y. Bayesian Parameter Inference and Model Selection by Population Annealing in Systems Biology. PLoS ONE. 2014;9(8):e104057 doi: 10.1371/journal.pone.0104057 25089832 10.1371/journal.pone.0104057 PMC4121267

[54] Lawson MJ, Drawert B, Khammash M, Petzold L, Yi TM. Spatial stochastic dynamics enable robust cell polarization. PLoS Comput Biol. 2013;9(7):e1003139 doi: 10.1371/journal.pcbi.1003139 23935469 10.1371/journal.pcbi.1003139 PMC3723497

[55] Bender A, Sprague GF. Yeast peptide pheromones, a-factor and alpha-factor, activate a common response mechanism in their target cells. Cell. 1986;47(6):929–937. doi: 10.1016/0092-8674(86)90808-1 3022943 10.1016/0092-8674(86)90808-1

[56] Dohlman HG, Thorner JW. Regulation of G protein-initiated signal transduction in yeast: paradigms and principles. Annual Review of Biochemistry. 2001;70:703–754. doi: 10.1146/annurev.biochem.70.1.703 11395421 10.1146/annurev.biochem.70.1.703

[57] Pruyne D, Bretscher A. Polarization of cell growth in yeast. I. Establishment and maintenance of polarity states. Journal of Cell Science. 2000;113(Pt 3):365–375. 10639324 10.1242/jcs.113.3.365

[58] Bourne HR, Sanders DA, McCormick F. The GTPase superfamily: a conserved switch for diverse cell functions. Nature. 1990;348(6297):125–132. doi: 10.1038/348125a0 2122258 10.1038/348125a0

[59] Epperson J. On the Runge Example. The American Mathematical Monthly. 1987;94:329–341. doi: 10.2307/2323093

[60] Chou CS, Nie Q, Yi TM. Modeling robustness tradeoffs in yeast cell polarization induced by spatial gradients. PLoS ONE. 2008;3:e3103 doi: 10.1371/journal.pone.0003103 21267054 10.1371/journal.pone.0003103 PMC3021495

[61] Chou CS, Bardwell L, Nie Q, Yi TM. Noise filtering tradeoffs in spatial gradient sensing and cell polarization response. BMC Syst Biol. 2011;5:196 doi: 10.1186/1752-0509-5-196 22166067 10.1186/1752-0509-5-196 PMC3268761

[62] Candes E, Romberg J. l1-magic: Recovery of sparse signals via convex programming; 2005. https://statweb.stanford.edu/~candes/l1magic/downloads/l1magic.pdf.

[63] Li S, Tso GK, Long L. Powered embrassing parallel MCMC sampling in Bayesian inference, a weighted average intuition. Computational Statistics & Data Analysis. 2017;115:11–20. doi: 10.1016/j.csda.2017.05.005

[64] Martino L, Elvira V, Luengo D, Corander J, Louzada F. Orthogonal parallel MCMC methods for sampling and optimization. Digital Signal Processing. 2016;58:64–84. doi: 10.1016/j.dsp.2016.07.013

[65] Solonen A, Ollinaho P, Laine M, Haario H, Tamminen J, Järvinen H. Efficient MCMC for climate model parameter estimation: parallel adaptive chains and early rejection. Bayesian Analysis. 2012;7(3):715–736. doi: 10.1214/12-BA724

[66] Rosenthal JS. Parallel computing and Monte Carlo algorithms. Far East Journal of Theoretical Statistics. 2000;4:207–236.

[67] Atkinson AC, Donev AN, Tobias RD.Optimum experimental designs, with SAS Oxford Statistical Science Series. Oxford University Press, Oxford; 2007.

[68] Yan L, Shin Y, Xiu D. Sparse Approximation using *ℓ*_1_ − *ℓ*_2_ Minimization and Its Application to Stochastic Collocation. SIAM J Sci Comput. 2017;39(1):A229–A254. doi: 10.1137/15M103947X

[69] Jenness DD, Spatrick P. Down regulation of the alpha-factor pheromone receptor in S. cerevisiae. Cell. 1986;46(3):345–353. doi: 10.1016/0092-8674(86)90655-0 3015412 10.1016/0092-8674(86)90655-0

[70] Bajaj A, Celić A, Ding FX, Naider F, Becker JM, Dumont ME. A fluorescent alpha-factor analogue exhibits multiple steps on binding to its G protein coupled receptor in yeast. Biochemistry. 2004;43(42):13564–13578. doi: 10.1021/bi0494018 15491163 10.1021/bi0494018

[71] Ventura AC, Bush A, Vasen G, Goldín MA, Burkinshaw B, Bhattacharjee N, et al Utilization of extracellular information before ligand-receptor binding reaches equilibrium expands and shifts the input dynamic range. Proc Natl Acad Sci U S A. 2014;111(37):E3860–9. doi: 10.1073/pnas.1322761111 25172920 10.1073/pnas.1322761111 PMC4169960

[72] Bendezú FO, Vincenzetti V, Vavylonis D, Wyss R, Vogel H, Martin SG. Spontaneous Cdc42 polarization independent of GDI-mediated extraction and actin-based trafficking. PLoS Biol. 2015;13(4):e1002097 doi: 10.1371/journal.pbio.1002097 25837586 10.1371/journal.pbio.1002097 PMC4383620

[73] Zi Z. Sensitivity analysis approaches applied to systems biology models. IET Systems Biology. 2011;5(6):336–346. doi: 10.1049/iet-syb.2011.0015 22129029 10.1049/iet-syb.2011.0015

[74] Sobol IM, Kucherenko S. Derivative based global sensitivity measures and their link with global sensitivity indices. Mathematics and Computers in Simulation. 2009;79(10):3009–3017. doi: 10.1016/j.matcom.2009.01.023

[75] Green PJ. Reversible Jump Markov Chain Monte Carlo Computation and Bayesian Model Determination. Biometrika. 1995;82(4):711–732. doi: 10.1093/biomet/82.4.711

[76] Andrieu C, Djurić PM, Doucet A. Model selection by MCMC computation. Signal Processing. 2001;81:19–37. doi: 10.1016/S0165-1684(00)00188-2

[77] Ohio Supercomputer Center. Ohio Supercomputer Center; 1987. http://osc.edu/ark:/19495/f5s1ph73.

