# Supplementary material for: Parameter uncertainty quantification using surrogate models applied to a spatial model of yeast mating polarization
Source: PLoS Comput Biol. 2018 May 29;14(5):e1006181. doi: 10.1371/journal.pcbi.1006181 (PMC5993324; doi:10.1371/journal.pcbi.1006181)
Supplement: S1 Table — Parameter values are taken from [37]. Values for kGa and kGd were estimated based on least-squares fit to time course and dose-response data. (PDF) [file pcbi.1006181.s006.pdf]

Parameter	Value
k_{RL}	2×10^{-3} nM^{-1}s^{-1}
k_{RLm}	10^{-2} s^{-1}
k_{Rs}	4 molecules per cell s^{-1}
k_{Rd0}	4×10^{-4} s^{-1}
k_{Rd1}	4×10^{-3} s^{-1}
k_{G1}	1 (molecules per cell)$^{-1}$s^{-1}
Gt	10000 molecules per cell
k_{Ga}	10^{-5} (molecules per cell)$^{-1}$s^{-1}
k_{Gd}	0.11 s^{-1}

S1 Table. Parameter values for Model 1. Parameter values are taken from [37]. Values for k_{Ga} and k_{Gd} were estimated based on least-squares fit to time course and dose-response data.
