# Supplementary material for: Parameter uncertainty quantification using surrogate models applied to a spatial model of yeast mating polarization
Source: PLoS Comput Biol. 2018 May 29;14(5):e1006181. doi: 10.1371/journal.pcbi.1006181 (PMC5993324; doi:10.1371/journal.pcbi.1006181)
Supplement: S2 Table — Experimental data for the given time points and α-factor levels from [37], and the resulting data. Output is the fraction of free Gβγ (Gbg/Gt). Data are given as mean ± standard deviation. (PDF) [file pcbi.1006181.s007.pdf]

Time course data			Dose-response data		
α-factor (L)	Time (T)	Output	α-factor (L)	Time (T)	Output
1000 nM	10 s	0.3504 ± 0.008	1 nM	60 s	0.06 ± 0.008
1000 nM	30 s	0.4 ± 0.004	2 nM	60 s	0.108 ± 0.02
1000 nM	60 s	0.368 ± 0.012	5 nM	60 s	0.2 ± 0.028
1000 nM	120 s	0.3868 ± 0.0148	10 nM	60 s	0.304 ± 0.024
1000 nM	210 s	0.3304 ± 0.016	20 nM	60 s	0.4 ± 0.012
1000 nM	300 s	0.242 ± 0.032	50 nM	60 s	0.408 ± 0.004
1000 nM	450 s	0.1748 ± 0.024	100 nM	60 s	0.412 ± 0.012
1000 nM	600 s	0.1948 ± 0.016			

S2 Table. Experimental data. Experimental data for the given time points and α-factor levels from [37], and the resulting data. Output is the fraction of free G$\beta\gamma$ (Gbg/Gt). Data are given as mean ± standard deviation.
