# Supplementary material for: Parameter uncertainty quantification using surrogate models applied to a spatial model of yeast mating polarization
Source: PLoS Comput Biol. 2018 May 29;14(5):e1006181. doi: 10.1371/journal.pcbi.1006181 (PMC5993324; doi:10.1371/journal.pcbi.1006181)
Supplement: S3 Table — Ranges for the kinetic parameters used for parameter estimation of all 8 parameters in Model 1 (heterotrimeric G-protein model). (PDF) [file pcbi.1006181.s008.pdf]

Parameter	Range
k_{RL}	$[10^{-4}, 10^{-2}]$
k_{RLm}	$[10^{-3}, 10^{-1}]$
k_{Rs}	$[10^{-1}, 10]$
k_{Rd0}	$[10^{-5}, 10^{-3}]$
k_{Rd1}	$[10^{-4}, 10^{-2}]$
k_{G1}	$[10^{-1}, 10]$
k_{Ga}	$[10^{-6}, 10^{-4}]$
k_{Gd}	$[10^{-2}, 1]$

S3 Table. Parameter ranges for Model 1. Ranges for the kinetic parameters used for parameter estimation of all 8 parameters in Model 1 (heterotrimeric G-protein model).
