# Supplementary material for: Parameter uncertainty quantification using surrogate models applied to a spatial model of yeast mating polarization
Source: PLoS Comput Biol. 2018 May 29;14(5):e1006181. doi: 10.1371/journal.pcbi.1006181 (PMC5993324; doi:10.1371/journal.pcbi.1006181)
Supplement: S4 Table — Sensitivity coefficients, in order of ascending magnitude, from sensitivity analysis of all 35 parameters in Model 2 using a 5th order surrogate polynomial fit to 5000 sample points. (PDF) [file pcbi.1006181.s009.pdf]

Parameter	Sensitivity	Parameter	Sensitivity
D_{B1m}	-0.000240521	$C42_t$	0.00831949
G_t	-0.00025359	D_{Gbg}	-0.0097571
D_{Ga}	-0.000704648	$B1_t$	0.0172396
D_{RL}	-0.000734672	k_{24d}	-0.0184795
D_{Gd}	-0.00106669	$C24_t$	0.0203875
k_{Ga}	0.00151801	k_{Cla4a}	-0.0210338
k_{Rs}	0.0015794	k_{Cla4d}	0.0247716
R_t	-0.00167628	k_{B1cm}	0.0463723
k_{Rd0}	0.00232993	k_{24cm0}	-0.0521665
D_{c24m}	-0.00233107	k_{B1mc}	-0.0550074
k_{G1}	0.00234184	D_{c42a}	-0.0557088
k_{24mc}	-0.00243174	h	0.056832
k_{RLm}	0.00348159	D_{c42}	0.0624607
k_{Rd1}	0.00401553	q	0.0744311
k_{Gd}	0.0040475	k_{42d}	-0.0962736
D_R	-0.00517784	k_{24cm1}	0.0999564
D_G	0.00608915	k_{42a}	0.110069
k_{RL}	-0.00789241		

S4 Table. Sensitivity coefficients for Model 2. Sensitivity coefficients, in order of ascending magnitude, from sensitivity analysis of all 35 parameters in Model 2 using a 5th order surrogate polynomial fit to 5000 sample points.
