# Supplementary material for: Parameter uncertainty quantification using surrogate models applied to a spatial model of yeast mating polarization
Source: PLoS Comput Biol. 2018 May 29;14(5):e1006181. doi: 10.1371/journal.pcbi.1006181 (PMC5993324; doi:10.1371/journal.pcbi.1006181)
Supplement: S1 Text — (PDF) [file pcbi.1006181.s010.pdf]

S1. Parameter sets

Model 1

Allowing all 8 kinetic parameters to vary, we obtained the parameter set P_{mean} by taking the mean of the parameter probability distributions from the MCMC chain. This parameter set and the maximum likelihood parameter set from [37] are given below.

Parameter	P_{mean}	P_{opt}
k_{RL}	2.7945×10^{-3}	2×10^{-3}
k_{RLm}	7.3044×10^{-3}	10^{-2}
k_{Rs}	1.1228	4
k_{Rd0}	9.0087×10^{-5}	4×10^{-4}
k_{Rd1}	2.4735×10^{-3}	4×10^{-3}
k_{G1}	9.0032×10^{-1}	1
k_{Ga}	1.1471×10^{-5}	10^{-5}
k_{Gd}	1.5609×10^{-1}	1.1×10^{-1}

Table A. Mean parameter set from MCMC parameter estimation for Model 1.

Model 2

After reducing the parameter count to 15 parameters, we are able to determine point estimates of the parameters by taking the mean of the probability distributions from MCMC. We also use simulated annealing to find an ‘optimal’ parameter set. Both of these parameter sets, as well as their corresponding model output polarization factors, are given below. The mean polarization factor of the data was 0.8733.

Parameter	MCMC mean	SA
k_{24cm0}	3.0320055×10^{-2}	4.5138694×10^{-2}
k_{24cm1}	7.6652079×10^{-3}	2.6129671×10^{-3}
k_{24d}	9.8590235×10^{-1}	6.9810380×10^{-1}
k_{42a}	1.9826224×10^{-4}	3.3996171×10^{-5}
k_{42d}	1.1113222×10^{-1}	2.3604239×10^{-2}
k_{B1cm}	1.5080301×10^{-4}	3.1423170×10^{-5}
k_{B1mc}	6.3228081×10^{-2}	6.5626614×10^{-2}
k_{Cla4a}	5.5002477×10^{-3}	1.2886983×10^{-2}
k_{Cla4d}	1.2111474×10^{-2}	1.1516911×10^{-2}
$C24_t$	1.8312125×10^{3}	1.1631549×10^{3}
$B1_t$	3.2136500×10^{3}	2.5837722×10^{3}
q	5.6447425×10^{1}	9.2959120×10^{1}
h	5.2838600	7.7413850
D_{c42}	1.2569088×10^{-2}	1.7240839×10^{-2}
D_{c42a}	7.8515533×10^{-3}	6.2231633×10^{-3}
Polarization factor	0.5722	0.6389

Table B. Parameter sets from MCMC parameter estimation and simulated annealing for Model 2.
