# Supplementary material for: Parameter uncertainty quantification using surrogate models applied to a spatial model of yeast mating polarization
Source: PLoS Comput Biol. 2018 May 29;14(5):e1006181. doi: 10.1371/journal.pcbi.1006181 (PMC5993324; doi:10.1371/journal.pcbi.1006181)
Supplement: S2 Text — (PDF) [file pcbi.1006181.s011.pdf]

S2. Numerical methods

In our numerical simulations, the cell membrane is simulated as a circle centered at the origin with radius 2 μm. The pheromone gradient is administered from the positive x direction. The computational domain is parametrized by $\alpha \in [0, 2\pi]$, where α denotes the angle from the negative x-axis. The surface diffusion of a quantity W on a circle is given by

$$\nabla_m^2 W = W_{ss}$$

where s is an arc length parameter, $ds^2 = dx^2 + dy^2$. Using the chain rule, this can be computed in terms of the parameter α as

$$W_{ss} = \frac{s'(\alpha)W_{\alpha\alpha} + s''(\alpha)W_{\alpha}}{(s'(\alpha))^3}.$$

The numerical method employed utilizes a second order finite difference discretization for the spatial derivatives, and an implicit Crank-Nicolson method (described below) for the temporal approximation. The method is second-order accurate in space and time. In our simulations, the spatial mesh consists of 400 equally spaced points and the time step is $\Delta t = 10^{-2}$. The code is written in Fortran.

Temporal approximation

Consider the problem $\dot{u} = F(u)$. Denote the solution u at time $t_n = n\Delta t$ by u_n. Then the problem can be approximated by

$$\frac{u_{n+1} - u_n}{\Delta t} = \frac{1}{2}(F(u_{n+1}) + F(u_n))$$

Moving unknowns to one side,

$$u_{n+1} - \frac{\Delta t}{2}F(u_{n+1}) = u_n + \frac{\Delta t}{2}F(u_n)$$

This requires the solution of a nonlinear equation. To solve the nonlinear system, we use the fixed point iteration:

1. Define $G(y) = \left(y - \frac{\Delta t}{2}F(y)\right) - \left(u_n + \frac{\Delta t}{2}F(u_n)\right)$.
2. Let $y_0 = u_n$.
3. Iterate $y_{k+1} = y_k - G(y_k)$ until convergence. Denote the final iterate by y.
4. Let $u_{n+1} = y$.

Since the fixed point iteration is not guaranteed to converge, we make the time-step adaptive in the following way: if the iteration number exceeds 20, reduce the current time-step by a factor of 2.
